# Multiscale-structured miniaturized 3D force sensors

**DOI:** 10.1038/s41563-026-02508-7

**Published:** 2026-02-18

**Authors:** Guolin Yun, Zesheng Chen, Zhuo Chen, Jinrui Chen, Binghan Zhou, Mingfei Xiao, Michael Stevens, Manish Chhowalla, Tawfique Hasan

**Affiliations:** 1 Cambridge Graphene Centre, University of Cambridge, Cambridge, UK; 2 Department of Modern Mechanics, University of Science and Technology of China, Hefei, China; 3 Institute of Humanoid Robots, University of Science and Technology of China, Hefei, China; 4 Department of Engineering, University of Cambridge, Cambridge, UK; 5 Department of Materials Science and Metallurgy, University of Cambridge, Cambridge, UK

**Keywords:** Mechanical and structural properties and devices, Electrical and electronic engineering

## Abstract

Flexible tactile sensors are pivotal for advancing neuroprosthetics, human–machine interactions and intelligent robotics. However, achieving highly sensitive tactile sensing to differentiate normal and tangential forces, particularly in mimicking the high-resolution multidimensional haptics of human fingers, remains a challenge. Here we propose a triaxial force microsensor array made from graphene–liquid-metal composites. Using anisotropic particle networks in microporous composites with pyramid geometries, we achieve normal–tangential force decoupling through multiscale structuring. Our approach offers exceptional sensitivity of 110 kPa^−1^ over a 500 kPa linear range (*R*^2^ > 0.998), with <2° force direction measurement deviation. The sensor array demonstrates force decoupling and slip detection via self-adjusted grasping of unknown objects. Our microsensor improves on the state of the art by an order of magnitude in size and detection limit, enabling 3D force sensing in micromanipulators and microrobots and unlocking advanced robotic dexterity.

## Abstract (web summary)

Tactile sensing to differentiate normal and shear forces remains a challenge. A force microsensor array based on graphene and a liquid-metal composites decouples normal–shear force sensing, achieving a 200 μm scale and a 0.9 μN force detection limit.

## Main

The human finger is equipped with four types of mechanoreceptor (RA, PC, SA-I and SA-II)^1–3^, enabling the perception of multidimensional tactile forces that include pressure, vibration, shear stress and tensile strain. The combination of these sensations enables complex tactile information processing at high spatial resolution that includes force direction, sliding and texture perception^4,5^, which is essential for our interaction with the environment and execution of daily tasks^6–8^. The development of miniaturized multidimensional tactile sensors that are comparable to those of human fingers is, therefore, imperative for advancing next-generation robots with dextrous manipulation capabilities^9–12^.

In recent years, various tactile sensors have been developed to replicate the three-dimensional (3D) tactile sensation of fingertips^13,14^. These sensors use different mechanisms to decouple normal and shear forces, including changes in resistance^15,16^, capacitance^17,18^, output voltage^19^, magnetic field^3,20,21^, air pressure^22^ or optical signal^23,24^. However, the complex and bulky structures required for optoelectronically or mechanically decoupled three-axis force sensors^25,26^ hinder their integration into typical robotic arms or prosthetic limbs^26,27^. Various sensing schemes for detecting sliding or surface texture also face challenges in identifying force directions^7,28–31^. Whereas advancements have been made with sensors using multilayer piezoresistive materials and patterned electrodes^32,33^, many still struggle with accurately identifying the direction of tangential forces or are limited to measuring them in fixed directions^19,33,34^. In some sensors that incorporate electrode arrays^15,17,35,36^, their deformation under normal and shear loading generates distinct output signals on the electrodes, enabling the calculation of three-axis forces via mathematically decoupled models. However, these proof-of-concept demonstrations are large, and they have poor linearity, low sensitivity or a narrow measurement range, rendering them unsuitable for application scenarios that demand miniaturization such as in micromanipulators^4,15,18,22^. Replicating high-resolution multidimensional haptics that even approach the human finger remains a formidable challenge.

We propose multiscale-structured force-sensor arrays based on graphene-synergized anisotropic porous conductive elastomers (APEs) to address these challenges. Our composite incorporates a hybrid filler comprising spiky nickel (Ni) particles, few-layer graphene (FLG) nanosheets and liquid metal (LM) microdroplets, forming a solid–liquid hybrid conductive network with LM droplets as deformable hubs and FLG sheets as bridges. The LM used here refers to eutectic gallium–indium (EGaIn), which combines the deformability of liquid with the high conductivity of metal^37,38^. By introducing a porogen during the preparation process and curing under a magnetic field, the composite combines an interconnected microporous structure and an anisotropic filler network to achieve high force sensitivity along the alignment direction. We further imitate the structure of the human epidermis to create pyramid sensor arrays with individual sensing units as small as 200 μm. The use of pyramid structures enables an extremely high linear sensitivity and a wide detection range. Integrated into a robotic gripper, we demonstrate high-precision real-time sensing of force magnitude and direction, slippage and roughness, offering critical capabilities for haptic devices such as prosthetics and micromanipulators, and thus advancing robotic dexterity in unpredictable or unknown environments.

## Preparation of the APE

**Fig. 1:**
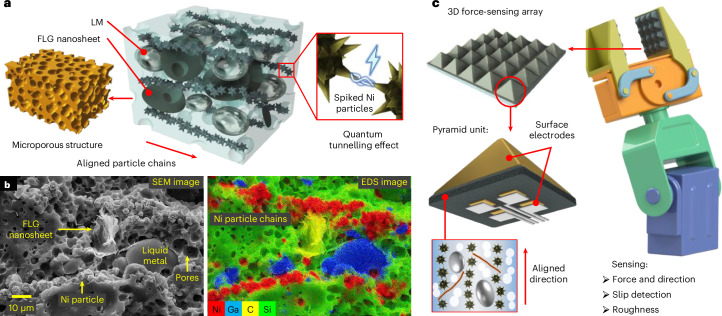
Multiscale structures of the APE sensor. **a**, Schematic showing the 3D composition and microstructures of the APE. **b**, SEM and EDS images of the cross-section of the APE sample. The distribution of Ni, FLG nanosheet, LM droplets and PDMS are represented by their characteristic elements of Ni, C, Ga and Si, respectively. The EDS image shows the aligned Ni particle chains. **c**, Schematic showing the APE 3D force-sensing array with pyramid surface structures mounted on a robotic manipulator.

Figure 1a illustrates the 3D microstructure of the APE, with its detailed preparation process given in Supplementary Fig. 1 and Methods. The LM–Ni particle mixture in polydimethylsiloxane (PDMS) is mixed with an FLG–PDMS dispersion and 1,2-propanediol (as a sacrificial porogen for micropore formation) at low speed. The mixture is then solidified under a uniform 500 mT magnetic field at 80 °C. APE samples are obtained by heating the cured composite at 140 °C for 3 h to evaporate the 1,2-propanediol through interconnected micropores (Supplementary Fig. 2).

Scanning electron microscopy (SEM) and energy-dispersive X-ray spectroscopy (EDS) images of the APE sample cross-section (Fig. 1b) show the Ni particles (with diameters of ~2–5 μm), FLG nanosheets (~20–40 μm), LM droplets (~15–30 μm) and pores (~3–8 μm). The Ni microparticles align along the magnetic field into chains, bridged by FLG nanosheets and LM droplets to construct an anisotropic conductive network within the microporous elastomer (Fig. 1b and Supplementary Fig. 3). At different scales, quantum tunnelling between the Ni particles^39–41^, FLG percolation network^42^, microporous structure and anisotropic particle network collaboratively improve the pressure sensitivity of the APE (detailed later). Mimicking the dermis structure, we establish a pyramidal protrusion array on the APE film (Fig. 1c). This structured film is coated with electrodes for pressure sensing and is encapsulated with an Ecoflex silicone rubber protective layer (Methods). This structure gives the APE sensor not only extraordinary sensitivity and range but also capabilities—including three-axis force sensing, slip detection and roughness estimation—that are beyond traditional sensors.

## Characterization and simulation of APE

We prepared the APE samples with varying compositions and processing methods, then characterized their electrical and mechanical properties to optimize the pressure sensitivity and sensing range. The pressure sensitivity (*S*) is defined as:

1 $$S=\frac{1}{{\sigma }_{0}}\frac{\Delta \sigma }{p}=\frac{1}{{\sigma }_{0}}\frac{\sigma -{\sigma }_{0}}{p},$$

where *σ*, *σ*_0_ and *p* denote the conductivity, initial conductivity and pressure, respectively. Specifically, *S* represents the ratio of the slope of the conductivity–pressure curve to the initial conductivity. The samples with a Ni/FLG/LM/PDMS mass ratio of 1.4/0.04/0.6/1 exhibit an optimal pressure-sensing performance (Supplementary Figs. 4–6). In addition, the FLG sheets are separately dispersed in PDMS at a low mixing speed to minimize breakage and maintain conductivity (Supplementary Figs. 7 and 8). Their structural integrity is preserved throughout subsequent tests and sensor operation (detailed later).

**Fig. 2:**
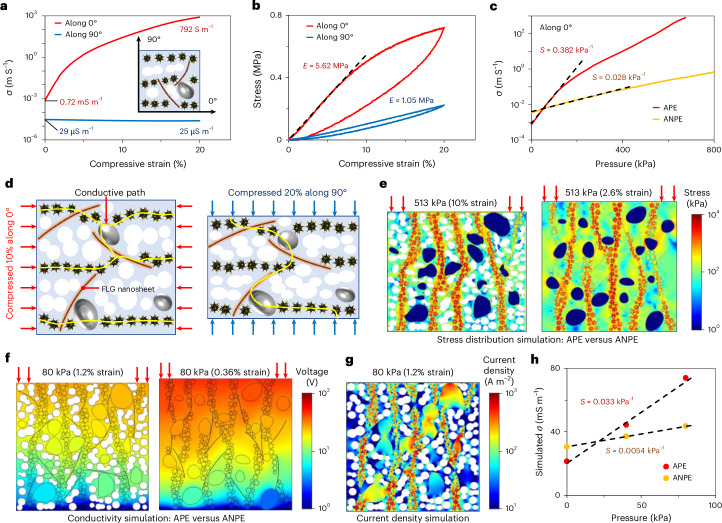
Electrical and mechanical properties of the APE. **a**,**b**, Conductivity–compressive strain curves (**a**) and stress–compressive strain curves (**b**) of the APE parallel (0°) and perpendicular (90°) to the alignment direction. Inset: the 0° and 90° directions defined based on the anisotropic filler network. **c**, Conductivity–pressure curves of the APE and the ANPE along 0°. **d**, Schematic illustrating the principle of conductivity change of the APE when compressed in different directions. **e**, Finite element simulations compare the deformation and stress distribution of the APE (left) and ANPE (right) under a pressure of 513 kPa. **f**, Finite element simulations compare the conductivity of the APE (left) and ANPE (right) under a pressure of 80 kPa. A larger electrical potential drop (red colour) corresponds to a greater resistivity. **g**, Current density simulations show conductive paths in the APE under pressure. **h**, Simulated conductivity of the APE and ANPE at different pressures.

The APE shows notable anisotropy attributed to the aligned Ni particle chains. The directions parallel and perpendicular to the chains are labelled 0° and 90°, respectively. The electrical conductivity–compressive strain and stress–compressive strain curves (Fig. 2a,b) show that the initial conductivity *σ*_0_ (724.8 μS m^−1^) and elastic modulus *E* (5.62 MPa) along 0° are, respectively, 24 times and 5.4 times those along the 90° direction. By adjusting the curing magnetic field strength, we achieve a six-orders-of-magnitude surge in conductivity at 20% compressive strain along 0°, while keeping the resistance along 90° stable (Supplementary Fig. 9). This insensitivity to lateral deformation avoids signal interference of the pressure sensors during stretching or curved-surface operation. Although the APE shows elastic hysteresis under cyclic loading (Supplementary Fig. 10), its conductivity–pressure response shows negligible hysteresis, avoiding the impact on pressure detection (Supplementary Fig. 11).

Our graphene-mediated network redesign, where FLG nanosheets bridge LM droplets and Ni chains, boosts the sensing performance and mechanical stability over previous LM composites^43,44^. The FLG’s high aspect ratio and flexibility reduces the need for metal fillers in conductive networks, vastly improving the composite’s flexibility and reliability. Crucially, FLG inclusion raises the conductivity by orders of magnitude and nearly triples the sensitivity, underpinning our anisotropic force transduction strategy in miniaturized 3D force sensors (Supplementary Fig. 12).

To evaluate the impact of the microporous structure on pressure sensing, we compared the APE with an anisotropic non-porous elastomer (ANPE) that was prepared without adding the porogen (Methods). According to Fig. 2c, the pressure sensitivity of the APE along 0° is 0.382 kPa^−1^, which is 13.6 times that of the ANPE. This is because the porous structure reduces the APE’s elastic modulus, enhancing deformation under pressure, especially at low pressures (Supplementary Fig. 13). The greater deformation of the particle network boosts conductivity, raising the pressure sensitivity.

Contrary to the sensitivity enhancement along 0°, the sensitivity of the APE along 90° falls below 10% of that for the ANPE (Supplementary Fig. 14). This contrast arises from conductive network changes under deformation (Fig. 2d). In the APE, FLG nanosheets and LM droplets form conductive paths within and between the Ni chains (Supplementary Fig. 15). Along 0°, the abundant paths bring high conductivity. Upon compression, reduced Ni particle contact resistance results in high sensitivity. Along 90°, however, transverse Ni chains and FLG/LM between them create serpentine conductive paths (Fig. 2d). When compressed, micropores absorb most of the deformation, stabilizing the contact resistance of the particles and the APE conductivity.

Finite element simulations validated the above hypothesis (for simulation settings see Supplementary Fig. 16), demonstrating considerably lower elastic modulus (Fig. 2e and Supplementary Fig. 17) and resistivity (Fig. 2f) for the porous composite under pressure. Current density simulations (Fig. 2g) visually depict the conductive paths in the APE, with a high current density in FLG and LM confirming their bridging function (clearer FLG current flow is shown in Supplementary Fig. 18). The simulated pressure sensitivity of the APE is an order of magnitude higher than that for the ANPE, validating the experimental findings (Fig. 2h).

## APE pyramid sensor unit

**Fig. 3:**
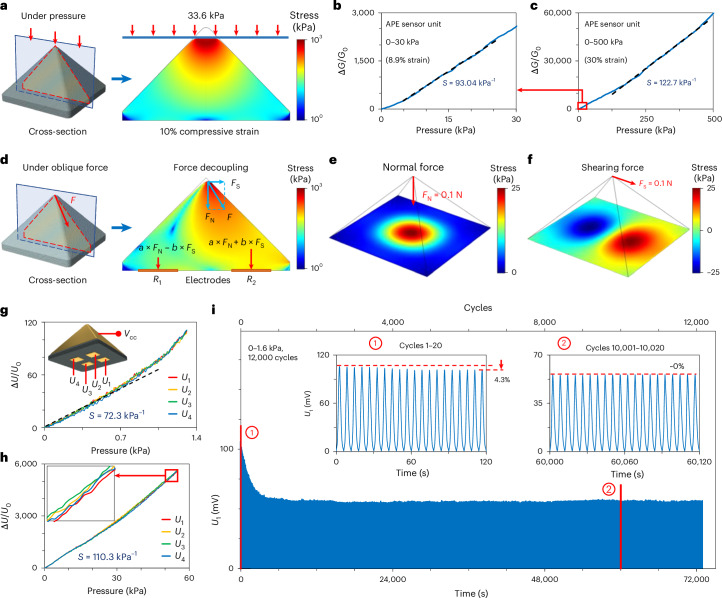
APE pyramid sensor unit. **a**, Simulated deformation and stress distribution in the longitudinal section of a pyramid sensor unit under vertical pressure. **b**,**c**, Curves showing the relative change in conductance (Δ*G*/*G*_0_) versus pressure for the APE sensor unit in the 0–30 kPa (**b**) and 0–500 kPa (**c**) pressure ranges. **d**, Simulated deformation and stress distribution in the longitudinal section of a sensing unit under an oblique force. *a*,*b*, constant coefficients related to sensor geometry and electrode positions; *R*_1_,*R*_2_, resistance measured at the electrodes on the bottom of the sensor unit. **e**,**f**, Simulated pressure distribution at the bottom of the APE sensor unit under normal force (**e**) and shear force (**f**). **g**,**h**, Curves showing the relative change in potential (Δ*U*/*U*_0_) versus pressure, as measured by 4 electrodes (*U*_1_–*U*_4_) on the bottom of the APE sensor unit in the 0–1.3 kPa (**g**) and 0–55 kPa (**h**) pressure ranges. The inset in **g** shows a schematic of the sensor unit; the inset in **h** shows an expansion of the data outlined by the red box. *V*_cc_, voltage at the common collector. **i**, Profile showing how the potential measured by the first electrode (*U*_1_) on the bottom of the sensor unit changes with time over 12,000 cycles of 0–1.6 kPa loading. Insets: the curves for the first 20 cycles (left) and for cycles 10,001–10,020 (right).

Inspired by the wavy interface of the human epidermis^45^ (Supplementary Fig. 19), we designed pyramid-shaped APE sensor units (with side lengths of 200 μm and 4 mm; Supplementary Fig. 20) to further improve the pressure sensitivity and enable normal–shear force decoupling. This type of sensor structure demonstrates substantial deformation and resistance changes under minuscule pressures, as illustrated via numerical simulations (Fig. 3a) and pressure–strain curves (Supplementary Fig. 21). Compared with a block APE that requires 80 kPa for 1.2% compression, the pyramid sensor unit achieves the same deformation and resistance change under compression of only 1.0 kPa, exhibiting an initial sensitivity enhancement of nearly two orders of magnitude. Further compression flattens the tip, increasing the modulus and pressure, thus providing a wide pressure-sensing range beyond previous high-sensitivity sensors.

Unlike conventional pyramid sensors that exploit tip geometry to adjust the contact area for sensitivity enhancement^46–49^, our design uses geometric compensation to overcome the inherent nonlinearity of the elastomer. The strategic placement of electrodes on the bottom of the sensor unit and a differential measurement scheme enable the convenient and precise decoupling of normal–shear forces. As shown in Fig. 3b,c, the sensor unit’s pressure–strain curve and nonlinear conductance–strain curve synergize to yield a highly linear conductance–pressure response (Supplementary Note 1). Using conductance to replace conductivity in equation (1), the sensor unit shows a pressure sensitivity of 93.0 kPa^−1^ and extreme linearity (*R*^2^ = 0.9991, where *R* is the coefficient of multiple correlation) below 175 kPa. Above 200 kPa, the pressure sensitivity rises to 122.7 kPa^−1^ while maintaining strong linearity (*R*^2^ = 0.9983). This extraordinary enhancement over a block-structured APE stems from the linear conductance–pressure response and stress concentration achieved by the pyramid geometry (Supplementary Fig. 22). Its exceptional linear sensitivity together with a range of up to 500 kPa surpass state-of-the-art flexible pressure sensors (Supplementary Fig. 23). This linearization, achieved through geometric compensation rather than material modification, offers a new approach to pyramid design for force sensors.

Moreover, our pyramid structure leverages its asymmetric stress distribution to decouple normal and shear forces (*F*_N_ and *F*_S_, respectively). Under an applied oblique force *F* at the sensor tip, shear force-induced asymmetric stress distribution occurs (see the simulated stress distribution in Fig. 3d). The average and difference in pressures at two symmetrical bottom points are proportional to the normal and shear forces, respectively. Figure 3e,f shows the bottom pressure distribution of a 4-mm-side-length sensor unit under a normal or shear force of 0.1 N. According to the pressure distribution data (Supplementary Fig. 24), we vapour-deposited four symmetrical gold electrodes onto the bottom of the sensor unit for pressure measurement (Supplementary Fig. 25). Whereas alternative electrode geometries (for example, triangular/pentagonal pyramids) can decouple forces, our design offers optimal robustness and electrode simplicity (Supplementary Fig. 25). Notably, the non-planar top electrode negligibly effects the internal potential distribution, thus preserving the force–response linearity across the full range of operation (detailed in Supplementary Fig. 26). As the force effects are linearly superimposable, normal and shear forces are calculable from the pressures on the four electrodes (detailed in the next section). Note that for forces applied at the side of the sensor unit, additional electrodes will be required for precise identification of the force magnitude/direction (Supplementary Fig. 27).

With read-out electronics (Supplementary Figs. 28–30), the output potential of the four bottom electrodes (*U*_1_–*U*_4_) increases with pressure. Figure 3g,h shows curves of the relative change in potential as a function of pressure recorded on the four electrodes under vertical pressures of 0–1.3 kPa and 0–55 kPa, respectively. Using equation (1), the pressure sensitivity at the *i*th electrode is defined as:

2 $${S}_{i}=\frac{1}{{U}_{i0}} \frac{\Delta {U}_{i}}{p}=\frac{1}{{U}_{i0}} \frac{{U}_{i}-{U}_{i0}}{p}\,\,(i=1,2,3,4),$$

where *U*_*i*_ is the potential of the *i*th electrode.

As the pressure is increased, the sensitivity of the sensor increases from 72.3 kPa^−1^ (~0–1 kPa) to 110.3 kPa^−1^ (>3 kPa) with high linearity (*R*^2^ = 0.997). Without tangential force, all four electrodes show overlapping relative potential curves due to their same pressure (a difference of <1.5% at ~50–55 kPa; Fig. 3h inset). In addition, the sensor exhibits high long-term stability under cyclic loading, with average response and recovery times of 19.5 ms and 27.2 ms, comparable to human skin^50^ (Supplementary Fig. 31). Figure 3i shows the potential–time curve of the first electrode over 12,000 cycles at 1.6 kPa. Although the maximum voltage decreases by 4.3% in the first 20 cycles due to rising resistance, it stabilizes after 400 cycles, with a deviation of <1.0% between the 800th and 12,000th cycles. Notably, the relative potential–pressure curve and pressure sensitivity remain constant throughout, reflecting exceptionally stability (Supplementary Fig. 32).

## 3D force sensing of the APE sensor

**Fig. 4:**
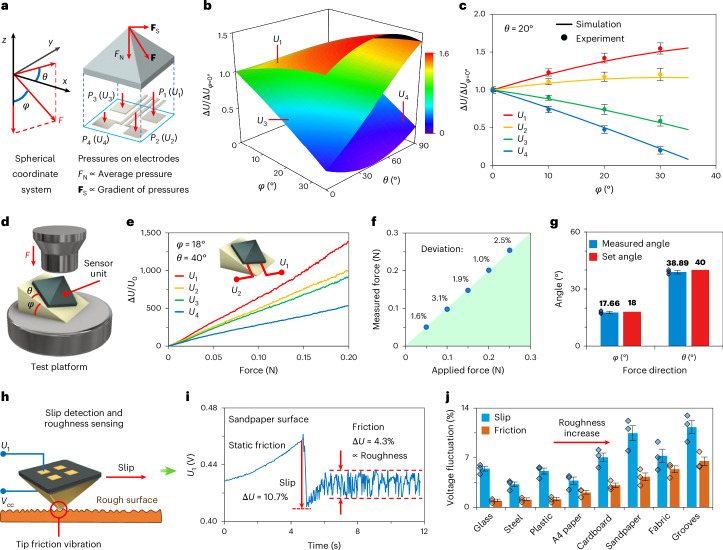
3D force sensing of the APE sensor unit. **a**, Decomposition of the oblique force in the spherical coordinate system (left) and different pressure distributions on the four electrodes at the bottom of the sensor unit (right). **b**, Simulated relative potential of the bottom electrodes under different force directions with a constant force magnitude. **c**, Comparison of simulation and experimental results for the relative potential versus *φ* curves of the four bottom electrodes when the direction of the shear force is fixed at 20°. Data are presented as the mean ± s.d. from *n* = 5 different sensors. **d**, Schematic showing the oblique force testing device of the APE sensor unit. **e**, Relative potential change versus force curves for the 4 electrodes of the sensor when the force direction is fixed at *φ* = 18° and *θ* = 40°. Inset: the position of the electrodes. **f**,**g**, Comparison of the force (**f**) and angles (**g**) measured by the sensor for different applied forces and force angles. Data are presented as the mean ± s.d. from *n* = 5 different tests. The green shaded area in **f** is equivalent to the *x* = *y* line to show the deviation of the measured forces from the applied forces. **h**, Schematic showing the sliding of the sensor unit on a rough surface. **i**, Potential–time curve of the first electrode when the sensor unit slides on sandpaper. **j**, Instantaneous changes and subsequent fluctuations in voltage when the sensor slides on different substrates. Data are presented as the mean ± s.d. from *n* = 3 different sensors.

Utilizing its force-decoupling capability, the APE pyramid sensor unit achieves high-precision identification of the spatial force direction, sliding detection and roughness estimation on the contact surface. The applied force *F* (in units of newtons) at the sensor tip is defined in spherical coordinates (Fig. 4a), where *φ* is the angle from the *z* axis and *θ* is the angle of the horizontal component (shear force) *F*_S_ from the *x* axis. As derived in Supplementary Note 2, the pressures on the four electrodes *P*_*i*_ for a 4 mm sensor unit are:

3 $$\begin{array}{c}{P}_{i}\,(\mathrm{kPa})=158.7\times F\times \cos (\varphi )\pm 166.9\times F\times \\ \sin (\varphi )[\cos (\theta )\pm \sin (\theta )]\,(i=1,2,3,4),\end{array}\,\,$$

with sign assignments of (+, +), (+ ,−), (−, −) and (−, +) for *P*_1_, *P*_2_, *P*_3_ and *P*_4_, respectively. Equation (3) is valid within the sensor’s linear range, ensuring measurement accuracy and protection of the FLG nanosheets (Supplementary Fig. 33).

As the change in electrode potential is proportional to the pressure, Fig. 4b plots the change in the relative potential of each electrode under different force directions at constant magnitude according to equation (3), where *θ* ranges from 0 to 90° (shear force in the first quadrant). For clarity, the data for the second electrode are omitted as the data for the second and third electrodes are symmetric about *θ* = 45° (Supplementary Fig. 34). Starting from *φ* = 0° (no shear force), *U*_1_ (the potential change on the first electrode) rises with shear force due to increased pressure *P*_1_. At *φ* = 35°, *θ* = 45°, *U*_4_ approaches zero, indicating no net force on the fourth electrode. Figure 4c compares the simulated and experimental changes in relative potential of the four electrodes as *φ* is increased (the force gradually tilts with a constant force magnitude) at *θ* = 20° (a constant shear force direction), and shows close agreement (deviation < 5.3%) despite increased error bars. Similar comparisons for *θ* = 0° and 40° show a maximum deviation of 6.1% (Supplementary Fig. 35).

According to equation (3), the force direction and magnitude can be calculated from the measured potentials *U*_*i*_:

4 $${V}_{i}=\frac{\Delta {U}_{i}}{{U}_{i0}}=\frac{{U}_{i}-{U}_{i0}}{{U}_{i0}}\,\,(i=1,2,3,4)$$

5 $$\theta ={\tan }^{-1}\left(\frac{{V}_{1}-{V}_{2}+{V}_{3}-{V}_{4}}{{V}_{1}+{V}_{2}-{V}_{3}-{V}_{4}}\right)$$

6 $$\varphi ={\tan }^{-1}\left(\frac{158.7}{166.9} \frac{\sqrt{2{({V}_{1}-{V}_{4})}^{2}+2{({V}_{2}-{V}_{3})}^{2}}}{{V}_{1}+{V}_{2}+{V}_{3}+{V}_{4}}\right)$$

7 $$|F|=\frac{({V}_{1}+{V}_{2}+{V}_{3}+{V}_{4})}{4\times {S}_{{F}_{{\rm{N}}}}\times \cos \varphi },$$

where *V*_*i*_ denotes the changes in relative potential of the electrodes at the bottom of the sensor unit and ${S}_{{F}_{{\rm{N}}}}$ is the normal force sensitivity calculated from Fig. 3h. Equations (5) and (6) show that the force direction depends solely on the relative magnitudes of the four electrode potentials.

Using the set-up shown in Fig. 4d (and detailed in Supplementary Note 3), we measure curves for the relative change in potential versus force for the four sensor electrodes at *φ* = 18°, *θ* = 40° (Fig. 4e). The force magnitudes and angles calculated from equations (4)–(7) deviated by <3.1% from the set values (Fig. 4f,g). Across 16 sensors from four batches, the maximum deviations of force, *φ* and *θ* that were measured are 5.7%, 2.2° and 5.2°, respectively, indicating excellent reproducibility and batch-to-batch consistency for scalable fabrication (Supplementary Fig. 36). The sensor also accurately tracked real-time 3D forces with varying magnitudes and directions (Supplementary Fig. 37 and Supplementary Video 1).

The sensor unit can also detect sliding with the contact surface and estimate the surface roughness. As the static friction coefficient exceeds the kinetic coefficient in most cases^51^, the onset of sliding abruptly reduces the shear force (the frictional force between the sensor unit and the contact surface) while the normal force remains relatively stable, causing a sudden change in electrode potentials. During sliding, the rough surface induces frictional vibrations on the sensor tip, causing electrical potential fluctuations related to the surface roughness. We take the sensor unit sliding on 120-grit sandpaper as an example (Methods). The first electrode bears the maximum pressure (Fig. 4h). Its potential *U*_1_ drops sharply by 10.7% at sliding initiation, followed by a fluctuation of ~4.3% during sliding (Fig. 4i). Note that the electrode potential on the side along the sliding direction increases, avoiding misjudgement from the overall force reduction (Supplementary Fig. 38). Figure 4j shows the instantaneous change and subsequent fluctuation amplitude of *U*_1_ when the sensor unit slides on various materials (their photos are shown in Supplementary Fig. 39). As the surface roughness increases from nearly zero (smooth glass) to 126 μm (for a groove structure), the voltage fluctuation increases from 0.95 to 6.5% (*R*^2^ = 0.96), highlighting its effectiveness in roughness estimation (Supplementary Fig. 40).

## APE sensor array for robotic manipulation

**Fig. 5:**
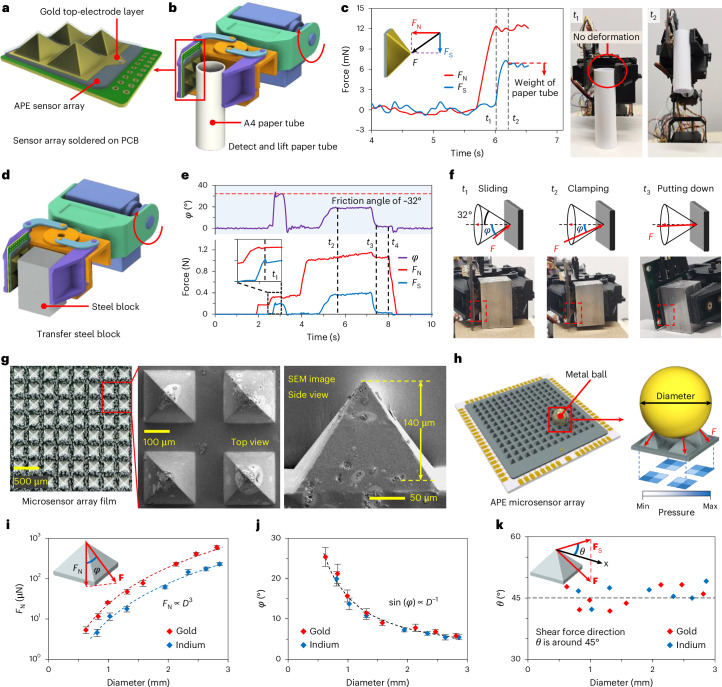
Demonstration of the APE sensor array. **a**, Schematic showing the APE force-sensor array soldered onto a PCB. **b**, Schematic illustrating the manipulator integrated with the APE sensor array grabbing a paper tube. **c**, Normal and tangential force–time curves (*F*_N_–*t* and *F*_S_–*t*, respectively) detected by the sensor when grabbing the paper tube. The photographs on the right show the paper tube being clamped (at *t*_1_) and lifted (*t*_2_). Inset: the definition of normal force *F*_N_ and tangential force *F*_S_. **d**, Schematic showing the manipulator grabbing a steel block. **e**, Normal and tangential force–time curves (bottom) and the force direction–time (*φ*–*t*) curve (top) detected by the sensor array when transferring the steel block. **f**, Photographs and measured forces on the steel block when it is sliding (*t*_1_), clamped (*t*_2_) and placed on the ground (*t*_3_). **g**, Photograph (left) and SEM images (middle and right) of the APE microsensor array. The white areas in the SEM images are due to charges on the PDMS matrix. **h**, Schematic showing the APE microsensor array bonded on a glass wafer with patterned electrodes (left) and the measured pressure distribution on the bottom of the four microsensor units in contact with a metal ball (right). **i**–**k**, The measured average normal force (**i**), force direction (**j**) and shear force direction (**k**) generated by gold and indium balls of different diameter (*D*) values. Data are presented as the mean ± s.d. for *n* = 5 derived from different sensor arrays. Insets: the definition of normal force *F*_N_ and force direction angles *φ* and *θ* in spherical coordinates.

We show an APE sensor array consisting of four 4 mm sensor units to demonstrate real-time 3D force-sensing abilities. The sensor array with vapour-deposited gold electrodes and an Ecoflex encapsulation layer was soldered onto a printed circuit board (PCB) (see Fig. 5a, Supplementary Fig. 41 and Methods for details) and assembled onto a manipulator gripper (Supplementary Fig. 42). Its output voltage signals were used for real-time feedback control of the pre-programmed manipulator (see the control circuit in Supplementary Fig. 43).

In a paper-tube-gripping demonstration (Fig. 5b), the sensor array exhibits high force sensitivity and a low detection limit. On contacting the paper tube, it detects a normal force of only 11 mN to avoid tube deformation and to control the manipulator for lifting the tube (*t*_1_ in Fig. 5c; detailed in Supplementary Fig. 44 and Supplementary Video 2). The sensor also accurately measures the weight of the paper tube (0.74 g) via the measured tangential force with a 3% error (*t*_2_ in Fig. 5c). By comparison, a commercial force sensor with a detection limit of 180 mN causes serious deformation of the paper tube during clamping (Supplementary Fig. 45 and Supplementary Video 3).

Operation in an unknown or untrained environment is critical for next-generation robotics. For this challenge, the detection abilities of the sensor for 3D force sensing and sliding are tested by transferring a steel block of unknown size and mass to a platform of unknown height (Fig. 5d). Supplementary Figure 46 and Supplementary Video 4 provide voltage signal–time curves and force-direction changes during the experiment. On clamping the steel block, the sensor promptly recognizes contact with the object (1.9 s in Fig. 5e). During lifting attempts, the sensor detects the increased shear force caused by gravity. When the steel block slips due to insufficient friction, the sensor instantly recognizes through a sudden ~5% drop in the shear force (*t*_1_ in Fig. 5e), subsequently tightening the gripper to clamp the block more securely. The force angle *φ* at *t*_1_, which is the friction angle, is approximately 32° (Fig. 5f). At *t*_2_, the steel block is lifted with a shear force of 0.38 N (equal to its gravity). On placement, the sensor identifies ground contact through a reduction in the shear force (*t*_3_ in Fig. 5e,f), triggering the gripper to release (*t*_4_ in Fig. 5e). By comparison, a manipulator equipped with a commercial force sensor fails to detect such slip (Supplementary Video 5) or ground contact (Supplementary Video 6).

The growing demand for miniaturized 3D force sensing, in particular for micro haptic devices and tactile spatial resolution that is comparable to human skin, underscores the potential of APE sensors. We prepared a microsensor array consisting of 200 μm sensor units to demonstrate this miniaturization capability (Fig. 5g, Supplementary Fig. 47 and Methods). The microsensor array film has a high spatial resolution of 300 μm and a surface pyramid height of 140 μm, ensuring that contact forces in practical tasks act mainly on the sensor tips (Supplementary Fig. 27). Each microsensor unit exhibits a high force sensitivity of 670 mN^−1^ and an extremely low detection limit of 0.9 μN, and good stability under long-term bending tests (Supplementary Fig. 48). Such a tiny size and low detection limit outperform state-of-the-art 3D force sensors by an order of magnitude, with potential for further miniaturization to 50 μm (see the comparison in Supplementary Fig. 49).

To demonstrate the 3D force-sensing capability of the microsensor array, we placed gold and indium balls (with diameters from 0.6 to 2.8 mm) sequentially on it. The forces acting on the four sensor units in contact with these metal balls are detected through photolithographically patterned electrodes on a glass wafer (Fig. 5h and Supplementary Fig. 50). Figure 5i–k presents the measured forces and directions generated by the gold and indium balls (the raw relative potential data are shown in Supplementary Fig. 51). Their mass and diameter can therefore be calculated to distinguish materials on the basis of density (detailed in Supplementary Fig. 52). In addition, by analysing the force-direction distribution, the sensor array has the potential to distinguish object geometries, such as spheres, plates and cylinders. This miniaturized design makes the sensor suitable for confined spaces in micromanipulators or microrobots.

## Outlook

Our development of a 3D force-sensor array using graphene-synergized anisotropic porous composites combines force-decoupled multiscale structures, and offers precise measurements of force magnitude and direction, along with the detection of sliding and estimation of the surface roughness. Through the innovative use of pyramid structures, our approach shows a sensitivity of 110 kPa^−1^ in a 500 kPa linear range, a deviation of less than 2° in measurement of the force direction deviation, a detection limit of 0.9 μN and the achievement of sensor units with a side length as small as 200 μm, surpassing state of the art by an order of magnitude. The sensor’s exceptional performance allows robotic arms to adaptively grasp objects of unknown size and weight, bringing us closer to replicating human-level tactile perception. Moreover, the potential to miniaturize the sensor units below 50 μm provides exciting possibilities for even finer spatial resolution, enabling sophisticated tasks in micromanipulators and microrobots. With further improvements in spatial resolution and repeatability, as well as the integration of temperature and humidity sensing, our sensor could achieve excellent performance in applications ranging from prosthetics to dextrous robotics.

## Methods

### Materials

The FLG nanoplatelets (thickness, 50 nm; diameter, ~25–45 μm) and 1,2-propanediol (98+% purity) were purchased from Sigma-Aldrich. The EGaIn (75 wt% gallium, 25 wt% indium) and Field’s metal alloy (51 wt% indium, 32.5 wt% bismuth, 16.5 wt% tin) were purchased from Magnametals. The spiky Ni microparticles (~2–5 µm in diameter) were purchased from APC Pure. The SYLGARD 184 Silicone Elastomer (PDMS) Curing Agent and SYLGARD 184 Silicone Elastomer (PDMS) Base were purchased from Univar Specialty Consumables. The Ecoflex 00-30 silicone rubber was purchased from 4.2 Bentley Advanced Materials. The AZ 5214E photoresist was purchased from MicroChemicals.

### Preparation of anisotropic porous elastomer

To prepare the APE, we first added the Ni particles (2.8 g), EGaIn (1.2 g) and liquid PDMS (1.2 g; PDMS base/curing agent mass ratio of 9:1) in a plastic cup with a mass ratio of Ni:EGaIn:PDMS of 1.4:0.6:0.6. These were then mixed using a flat plastic stick (2 × 5 mm cross-section, attached to an electric stirrer) at 350 rpm for 5 min. The FLG nanosheets (0.08 g) were then mixed with PDMS (0.8 g; FLG/PMDS mass ratio of 0.04:0.4) using the electric stirrer at 120 rpm for 5 min. The two mixtures were then combined with 1,2-propanediol (1.2 g) at 120 rpm for 5 min. Next, the obtained mixture (Ni/EGaIn/FLG/1,2-propanediol/PDMS mass ratio of 1.4/0.6/0.04/0.6/1) was poured into a 3D-printed plastic mould (material: thermoplastic polyurethane, TPU 95A) and solidified in a uniform magnetic field of 500 mT at 80 °C for 12 h. Finally, the cured APE samples were removed from the mould and heated in an oven at 140 °C for 3 h to remove the 1,2-propanediol. The final APE sample has a porosity of 27.9% and a Ni/EGaIn/FLG/PDMS volume ratio of 0.154/0.092/0.017/1.

### Preparation of other composite samples

The methods for preparing the ANPE and other isotropic porous or non-porous composites are the same as that of the APE. For the ANPE, the only differences are the absence of 1,2-propanediol and the solvent evaporation step after sample curing. As for the isotropic composites, the only difference between them and the APE or ANPE is that they were not solidified under an applied magnetic field.

### Preparation of the APE sensor unit and sensor array

To fabricate the 4 mm APE sensor unit and array, we first printed a pyramid-shaped mould using an Ultimaker S5 3D printer using the TPU 95A filament with a precision of 0.06 mm for curing the sensor. The 3D-printed masks were then used to evaporate electrodes on the top and bottom of the sensors using an electron-beam evaporator. A 5-nm-thick layer of chromium was first evaporated on the APE surface, and then a 30-nm-thick layer of gold was evaporated. The function of the chromium intermediate layer is to enhance the adhesion of the electrode to avoid peeling. Detailed electrode dimensions and their distribution are provided in Supplementary Figs. 25 and 41. The gold-plated electrode on the top of the sensor array was then drop-cast with an Ecoflex layer to encapsulate and protect the electrode from wear. When mounted on the gripper of a robot manipulator, the sensor array is soldered onto a self-designed PCB to ensure stable electrical contact. The electrodes on the PCB correspond to the bottom electrodes of the sensor array. The top electrode of the sensor array is also soldered to the PCB via holes. Field’s metal alloy with a melting point of 61 °C is used as solder to prevent damage to the sensor and electrodes at high temperatures. Note that we did not use LM solder because it is prone to leakage and cannot provide a secure connection (as detailed in Supplementary Fig. 53).

To fabricate the APE microsensor array with sensor a unit side length of 200 μm, a silicon wafer with an anisotropically etched pyramid pit array was used as the mould. We used AZ 5214E photoresist to laser-write a mask on a glass wafer and evaporate the electrode pattern (5 nm chromium and 100 nm gold) as the bottom electrode of the microsensor array (see Supplementary Fig. 50 for details). The reason for using the glass wafer is to facilitate alignment of the electrode patterns and microsensor array under the microscope. The microsensor array and the glass wafer were plasma-treated and then bonded together to achieve a stable electrical contact. After aligning the microsensor array and the bottom patterned electrodes, a 100-nm-thick gold layer was deposited on top as the top electrode. The assembled microsensor array was connected to an Arduino Uno Rev3 board through spring pin connectors. The rest of the preparation process is the same as for the 4 mm APE sensor array.

### Experimental equipment and set-up

An FEI Quanta 3D FEG dual-beam electron microscope was used to obtain the SEM and corresponding EDS images of the composite in Fig. 1b and Supplementary Fig. 3, all other SEM images were obtained using a Magellan 400 SEM instrument. The COMSOL Multiphysics 5.2 software package was used for finite element numerical simulation. An UltiMaker S5 3D printer was used to print the plastic moulds for curing the APE samples and sensors, the masks for electrode evaporation and the plastic parts in the testing device (Fig. 4d).

An Instron 68TM-50 Universal Testing System was used to compress the composite samples (5 × 5 × 5 mm) and sensors at a speed of 5% per min to measure their stress–compressive strain curves. Unless otherwise specified, all strains in this Article and its Supplementary Information refer to compressive strains. A Keithley 2400 Standard Series Source Measure Unit with a resistance range of 1 GΩ was used to measure the sample resistance. The final resistance is the measured resistance minus the internal resistance of the circuit with no sample present. All tests involving composite materials and sensor samples were performed at least five times to ensure statistical reliability. For all tests that include error bars, the values of the error bars represent the standard deviation calculated from five measurements. Unless otherwise specified, each experimental group was tested with five different samples prepared from the same batch. For sensor array testing, the interval between different sensor unit tests was 10 s. For the composite material with the optimal ratio, we tested more than 40 sensor units with a 4 mm size. The static resistance of these 4 mm units ranges from 2.7 to 3.4 MΩ, with a variation of less than 20%. For the 200–μm sensor units, more than 50 units were tested with a resistance variation of less than 30% (14.5–20.1 MΩ). These variations are primarily due to differences in the composite batches and the vapour-deposited electrodes. Notably, sensors with different static resistances exhibit identical relative resistance changes and output voltage responses under load. All sensors in the array were automatically calibrated on the basis of their static resistance before use, ensuring consistent relative voltage–force curves.

A Microtech LW-405B+ laser writer was used to photo-etch electrode the masks on glass and silicon wafers. An electron-beam evaporator (PVD 200 Pro, Kurt J. Lesker) was used to evaporate/deposit the gold electrodes on the APE samples and sensors. An Arduino Uno Rev3 board was used to control the sensor measurement circuit. An ADS1115 16-bit analogue-to-digital converter and an MCP3008 eight-channel analogue-to-digital converter were also used in this circuit. A self-designed PCB with an electrode array was used to fix the APE sensor on it for measurement. The sensor was soldered on the PCB at a low temperature of 80 °C using the Field’s metal (indium–bismuth–tin alloy, melting point 62 °C) to avoid burning the APE. To measure the sliding of the sensor unit on different substrates, the sensor unit was fixed on the PCB under a pressure of 5 kPa that was applied using the Instron testing machine. The substrate was clamped between the sensor tip and the jig of the testing machine and pulled by a screw guide at 2 mm s^−1^. A Bruker Dektak XT stylus profilometer was used to measure the surface roughness of various substrates. An AL5A 4 Degrees of Freedom (4DOR) robotic arm controlled by a BotBoarduino used for demonstration of theAPE sensor array was purchased from RobotShop.

## Online content

Any methods, additional references, Nature Portfolio reporting summaries, source data, extended data, supplementary information, acknowledgements, peer review information; details of author contributions and competing interests; and statements of data and code availability are available at 10.1038/s41563-026-02508-7.

## Supplementary information

Supplementary Information Supplementary Notes 1–3, Figs. 1–53, refs. 1–30 and titles of Videos 1–6.

Supplementary Video 1 Real-time 3D force sensing of the APE sensor unit.

Supplementary Video 2 The A4-paper-tube-gripping demonstration of a robotic arm equipped with an APE sensor array.

Supplementary Video 3 The A4-paper-tube-gripping demonstration of a robotic arm equipped with a commercial force sensor.

Supplementary Video 4 The steel-block-transferring demonstration of a robotic arm equipped with an APE sensor array.

Supplementary Video 5 The failure to detect the sliding of the steel block of a robotic arm equipped with a commercial force sensor.

Supplementary Video 6 The steel-block-transferring demonstration of a robotic arm equipped with a commercial force sensor.

## Data Availability

All data are available in the article and its Supplementary Information. The data used in the figures are publicly accessible through Apollo, the University of Cambridge repository, at 10.17863/CAM.124842 (ref. ^52^). Extra data are available from the corresponding author upon request.
