# Supplementary material for: Multiscale-structured miniaturized 3D force sensors
Source: Nat Mater. 2026 Feb 18;25(6):1011–9. doi: 10.1038/s41563-026-02508-7 (PMC13236590; doi:10.1038/s41563-026-02508-7)
Supplement: Supplementary file 1 — Supplementary Notes 1–3, Figs. 1–53, refs. 1–30 and titles of Videos 1–6. [file 41563_2026_2508_MOESM1_ESM.pdf]

nature materials

Supplementary information https://doi.org/10.1038/s41563-026-02508-7

Multiscale-structured miniaturized 3D force sensors

In the format provided by the authors and unedited

Supplementary Information

This file includes:

Supplementary Notes 1 to 3
Supplementary Figs. 1 to 53
References (*S1–S30*)

Other Supplementary Information for this manuscript include the following:

Supplementary Videos 1 to 6

Supplementary Note

Supplementary Note 1. Mechanistic Analysis of Linear Response Achievement.

Conventional pyramid-based sensors suffer from compromised linearity due to contact area saturation at high loads. While the expanding tip contact area under pressure grants high initial sensitivity, this geometric effect diminishes as the contact area approaches saturation, leading to severe sensitivity decay in high-pressure regimes*(46-49)*.

Our pyramid design overcomes this limitation through strategic coupling between intentionally engineered nonlinearities:

(1) Material nonlinearity: The composite exhibits exponential conductivity growth with strain (as shown in Fig. 2A), enhancing sensitivity at large deformations.

(2) Geometric nonlinearity: The pyramidal geometry creates a strain-hardening pressure response, counterbalancing the material's nonlinear conduction.

As illustrated in Fig. 3, B and C, the synergistic interaction between these two nonlinearities - where geometric strain-hardening compensates the increased sensitivity of composites - produces a linear conductivity-pressure relationship in 500 kPa range. This innovative approach transforms conventional trade-offs between sensitivity and linearity into complementary effects, which is different from conventional pyramid-based sensor design.

Supplementary Note 2. The average pressure on the bottom electrodes of the sensor unit.

According to the discussion in Supplementary Fig. 24, for sensor unit with a side length of 4 mm, the pressure on the four bottom electrodes in Fig. 4a is equal to 15.87 kPa under a normal force of 0.1 N. Under a tangential force of 0.1 N applied along the x-axis, the pressure on electrodes 1 and 2 (P_1 and P_2) is 16.69 kPa, and the pressure on electrodes 3 and 4 (P_3 and P_4) is -16.69 kPa. An analogy can

be made for the case of shear force applied along the y-axis. Under small force, APE can be regarded as a linear elastic material, and the bottom surface pressure distribution under different forces can be superimposed. Based on this, we can get the average pressure $P_1 \sim P_4$ on the four bottom electrodes under the force in Fig. 4a (the unit of force $\boldsymbol{F}$ is N):

$$P_1(kPa) = 158.7 \cdot \boldsymbol{F} \cdot \cos(\varphi) + 166.9 \cdot \boldsymbol{F} \cdot \sin(\varphi)\,[\cos(\theta) + \sin(\theta)] \quad \text{(S1)}$$

$$P_2(kPa) = 158.7 \cdot \boldsymbol{F} \cdot \cos(\varphi) + 166.9 \cdot \boldsymbol{F} \cdot \sin(\varphi)\,[\cos(\theta) - \sin(\theta)] \quad \text{(S2)}$$

$$P_3(kPa) = 158.7 \cdot \boldsymbol{F} \cdot \cos(\varphi) - 166.9 \cdot \boldsymbol{F} \cdot \sin(\varphi)\,[\cos(\theta) - \sin(\theta)] \quad \text{(S3)}$$

$$P_4(kPa) = 158.7 \cdot \boldsymbol{F} \cdot \cos(\varphi) - 166.9 \cdot \boldsymbol{F} \cdot \sin(\varphi)\,[\cos(\theta) + \sin(\theta)] \quad \text{(S4)}$$

Supplementary Note 3. Experimental setup for applying oblique force to APE sensor unit.

Figure 4d shows the schematic diagram of the oblique force testing device of a APE sensor unit with a side length of 4 mm. The sensor unit is mounted on a 3D printed tilted platform and compressed by the tensile tester. The potential-time curves on four bottom electrodes (recorded by Arduino UNO) and force/displacement-time curves (recorded by the tensile tester) are recorded during the test. The angle between the platform slope and the horizontal plane is the angle φ between the force $\boldsymbol{F}$ and the z-axis in Fig. 4a, while the angle between the sensor unit and the edge of the platform is the angle θ between the shear force $\boldsymbol{F_S}$ and the x-axis. Therefore, this experimental setup can conveniently adjust the φ and θ to test the force response curve of the sensor unit.

Supplementary Figures

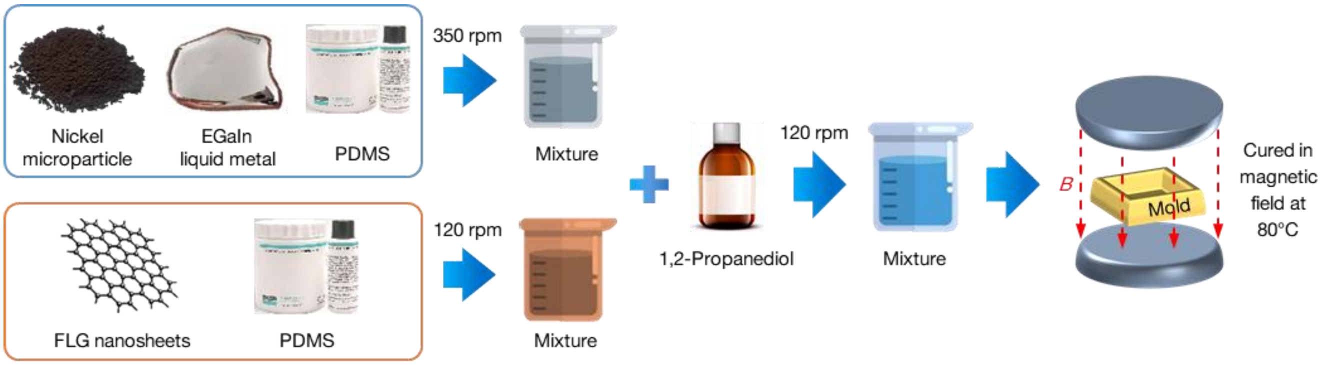

Supplementary Fig. 1.

Preparation methods of APE. See the detailed process in Methods.

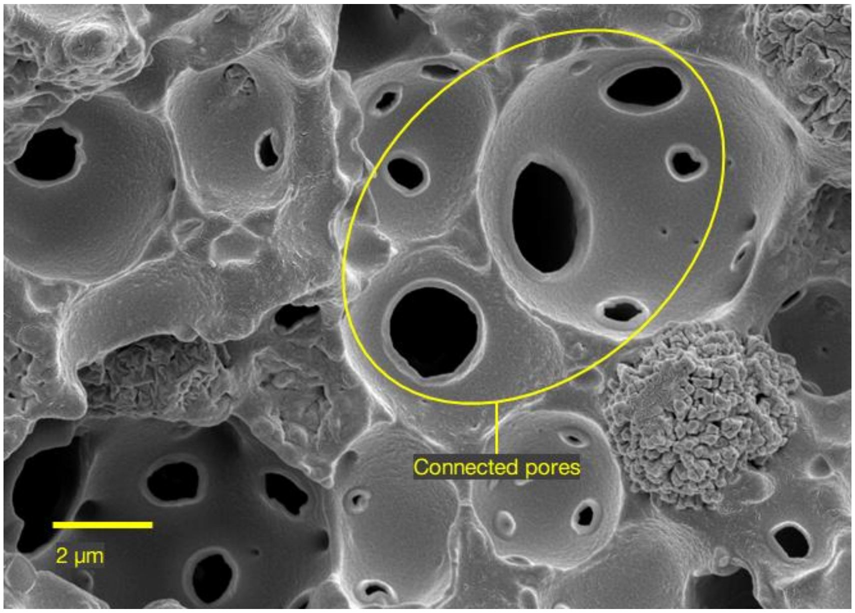

Supplementary Fig. 2.

The interconnected micropores in APE.

The SEM image given in Supplementary Fig. 2 shows the interconnected micropores structures in APE samples. Due to its high volume fraction, the porogen (1,2-Propanediol) forms contacted micro-droplets in the composite mixture during mixing process. After cured in oven, these droplets remain contacted in cured APE samples. During the high temperature heating process, the porogen droplets are evaporated and escape the APE sample from these connected pores, leaving behind an interconnected microporous structure. The diameters of these pores are 3~8 µm.

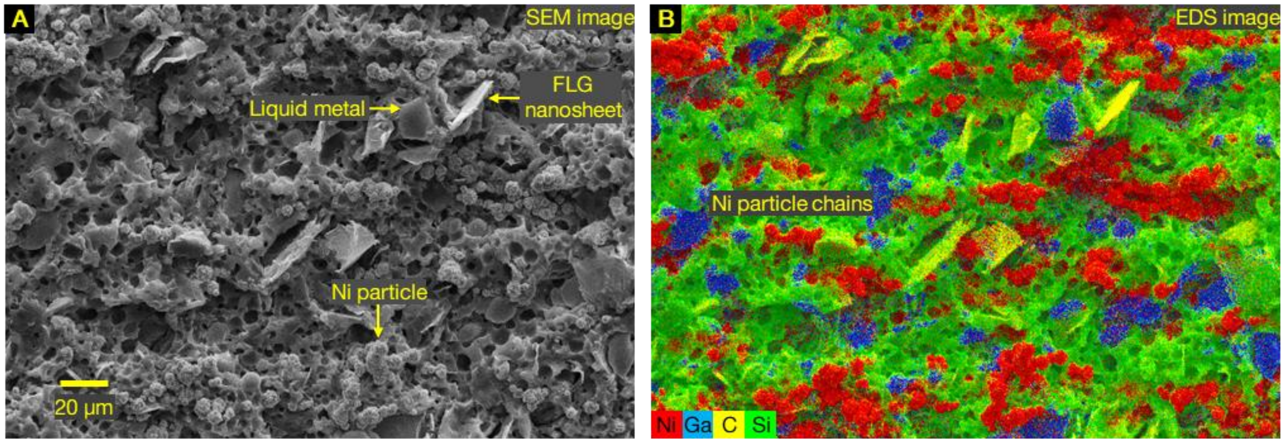

Supplementary Fig. 3.

The (**A**) SEM and (**B**) EDS images of the cross-section of APE sample with lower magnification, which shows the aligned Ni particle chain structure as well as the few layer graphene (FLG) nanosheets and LM droplets bridging them. The distribution of Ni, FLG nanosheet, LM droplets, and PDMS are represented by their characteristic elements of Ni, C, Ga and Si, respectively.

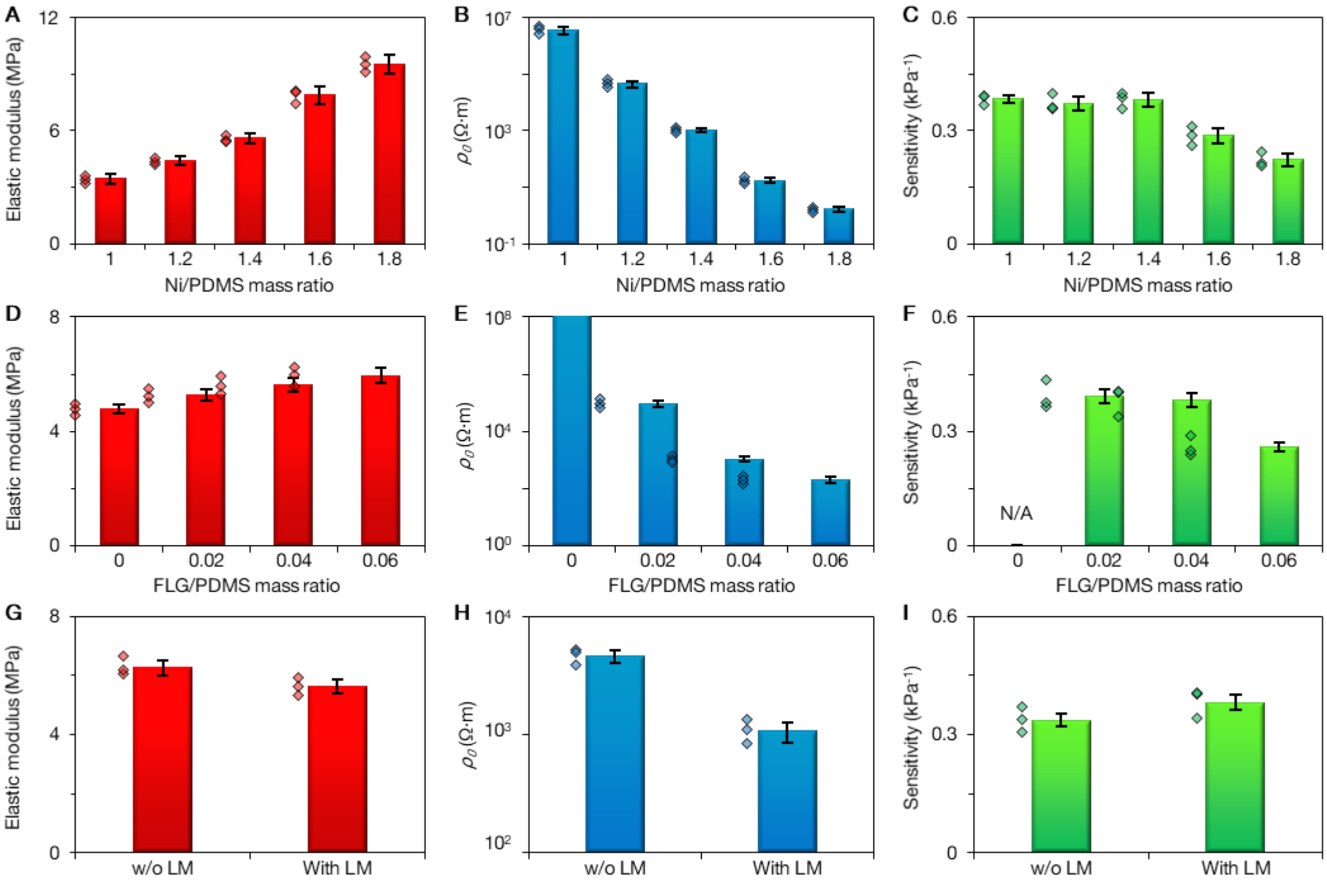

Supplementary Fig. 4.

Effects of (**A-C**) Ni content, (**D-F**) FLG content, and (**G-I**) LM content on the elastic modulus, initial resistivity, and initial pressure sensitivity of APE. For all error bars, n = 3 derived from different APE samples, data are presented as mean values +/- SD.

To investigate the influence of the conductive filler contents on the pressure sensing performance of APE, we prepare a set of APE samples with different Ni, FLG, and LM contents and compare their elastic modulus, initial resistivity, and initial pressure sensitivity. We initially selected an Ni/FLG/LM/PDMS mass ratio of 1.6/0.02/0.6/1 as the starting formulation. After conducting numerous controlled experiments, we found that the optimal ratio for pressure sensing performance is 1.4/0.04/0.6/1. In each set of controlled variable experiments, the other components were kept fixed at the above optimal filler/PDMS mass ratio. Since the surface structure has not yet been created on APE samples, their conductivity increases nonlinearly with pressure. We bring the conductivity at 80 kPa into Equation (1) to calculate the initial pressure sensitivity S.

As the Ni content increases, the elastic modulus and electrical conductivity of APE rise unsurprisingly (Supplementary Fig. 4, A and B). High Ni content significantly increases the stiffness of APE and hinders its deformation and resistance reduction under pressure, resulting in lower pressure sensitivity. However, the pressure sensitivity does not change much (around 0.38 kPa^{-1}) as the Ni/PDMS mass ratio increases from 1 to 1.4 (Supplementary Fig. 4C). This is because at low Ni content, the conductive particle network in APE is sparse and is less likely to form a large number of conductive paths under deformation, resulting in an insignificant reduction in resistance. When the Ni/PDMS mass ratio exceeds 1.4, the conductive particle paths in APE are dense and thus result in high initial conductivity. This makes the conductive paths more likely to saturate upon compression and limits the room for resistance to drop, resulting in significantly reduced sensitivity (0.223 kPa^{-1} for a Ni/PDMS mass ratio of 1.8).

A similar pattern can be found for the FLG content, except that the modulus of APE is less affected by the FLG content because FLG nanosheets are much more flexible than Ni particles and have a much lower volume fraction in the composite (Supplementary Fig. 4D). Note that the FLG-free composite is electrically insulating (resistivity > 100 MΩ·m), demonstrating the irreplaceable role of FLG in enhancing conductivity and reducing the Ni content required (Supplementary Fig. 4E). For similar reasons as in the case of Ni particles, the pressure sensitivity of APE fluctuates around 0.38 kPa^{-1} when the FLG/PDMS mass ratio is lower than 0.04, and drops by 32% to 0.259 kPa^{-1} as the FLG/PDMS mass ratio increases to 0.06 (Supplementary Fig. 4F). due to an order of magnitude lower initial resistivity and more stable initial resistance (shorter error bars), APE with a FLG/PDMS mass ratio of 0.04 exhibits better electrical properties than the case of 0.02.

In addition, liquid metal is also an integral part of the conductive network in APE. Although FLG nanosheets can bridge Ni particle chains to enhance conductivity, excess FLG can severely weaken the pressure sensitivity of the composite (Supplementary Fig. 4F). Therefore, the introduction of LM droplets can not only significantly improve the conductivity of APE by more than two orders of magnitude (Supplementary Fig. 4H), but also reduce the elastic modulus and enhance pressure sensitivity by ~14% (Supplementary Fig. 4I).

In summary, we set the Ni/FLG/LM/PDMS mass ratio at 1.4/0.04/0.6/1 to achieve the best pressure sensing performance of APE.

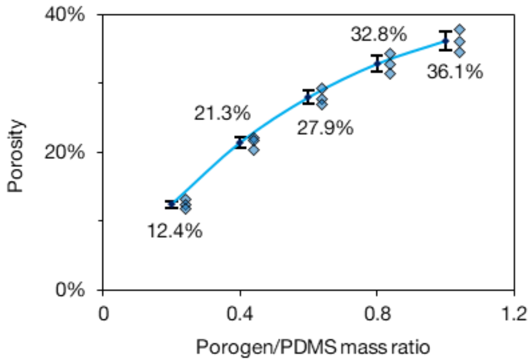

Supplementary Fig. 5.

The porosity of APE samples with different porogen/PDMS mass ratio. For all error bars, n = 3 derived from different APE samples, data are presented as mean values +/- SD.

Because composites shrink during high-temperature evaporation of the porogen, it is difficult to calculate their porosity according to the volumes of porogen and other raw materials in APE. We speculate that this is due to the shrinkage stresses occurring within the porous PDMS at high temperature. Therefore, we calculated the porosity of APE using the mass difference method. Specifically, we measured the mass of non-porous ANPE and porous APE composites with the same volume, recorded as M_1 and M_2, respectively. Then the porosity of the APE is: Porosity = $1-M_2/M_1$. The calculated porosity of APE for varying Porogen/PDMS mass ratios is given in Supplementary Fig. 5.

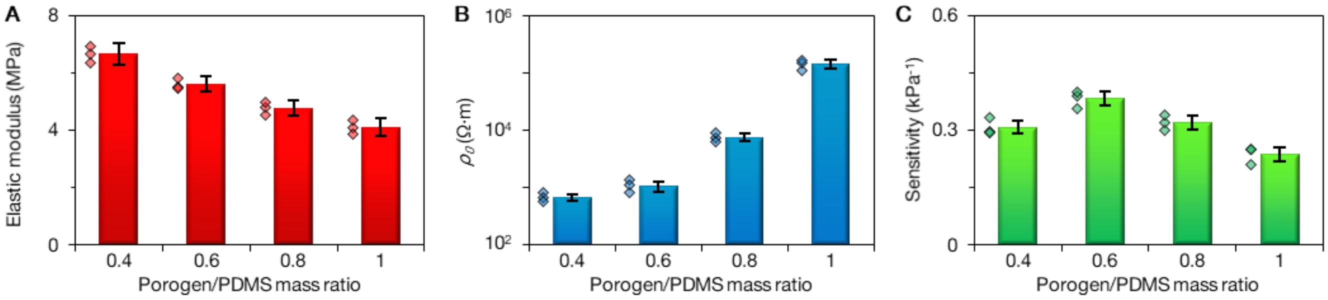

Supplementary Fig. 6.

Effects of porogen/PDMS mass ratio (porosity) on the (**A**) elastic modulus, (**B**) initial resistivity, and (**C**) initial pressure sensitivity of APE. For all error bars, n = 3 derived from different APE samples, data are presented as mean values +/- SD.

As the porosity (positively related to the porogen/PDM mass ratio) increases, the elastic modulus of APE decreases (Supplementary Fig. 6A), which theoretically contributes to enhanced pressure sensitivity. However, excessive porosity will destroy the conductive particle network in the composite, resulting in significantly reduced conductivity and electrical stability (Supplementary Fig. 6B). These micropores will also hinder the formation of conductive paths under pressure, which in turn weakens the pressure sensitivity of APE (Supplementary Fig. 6C). After investigation, APE with a porogen/PDM mass ratio of 0.6 (porosity of 27.9%) exhibits low resistivity (~1 kΩ·m) and the highest sensitivity (~0.38 kPa^{-1}).

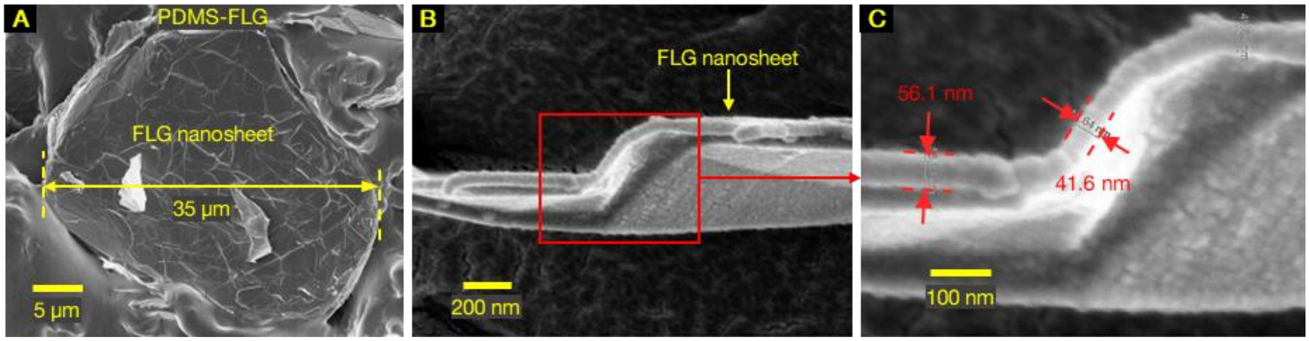

Supplementary Fig. 7.

The SEM images of FLG nanosheets. The signal layer FLG nanosheets generally have a thickness of ~50 nm and a diameter above 30 µm, showing a diameter-to-thickness ratio typically exceeds 600.

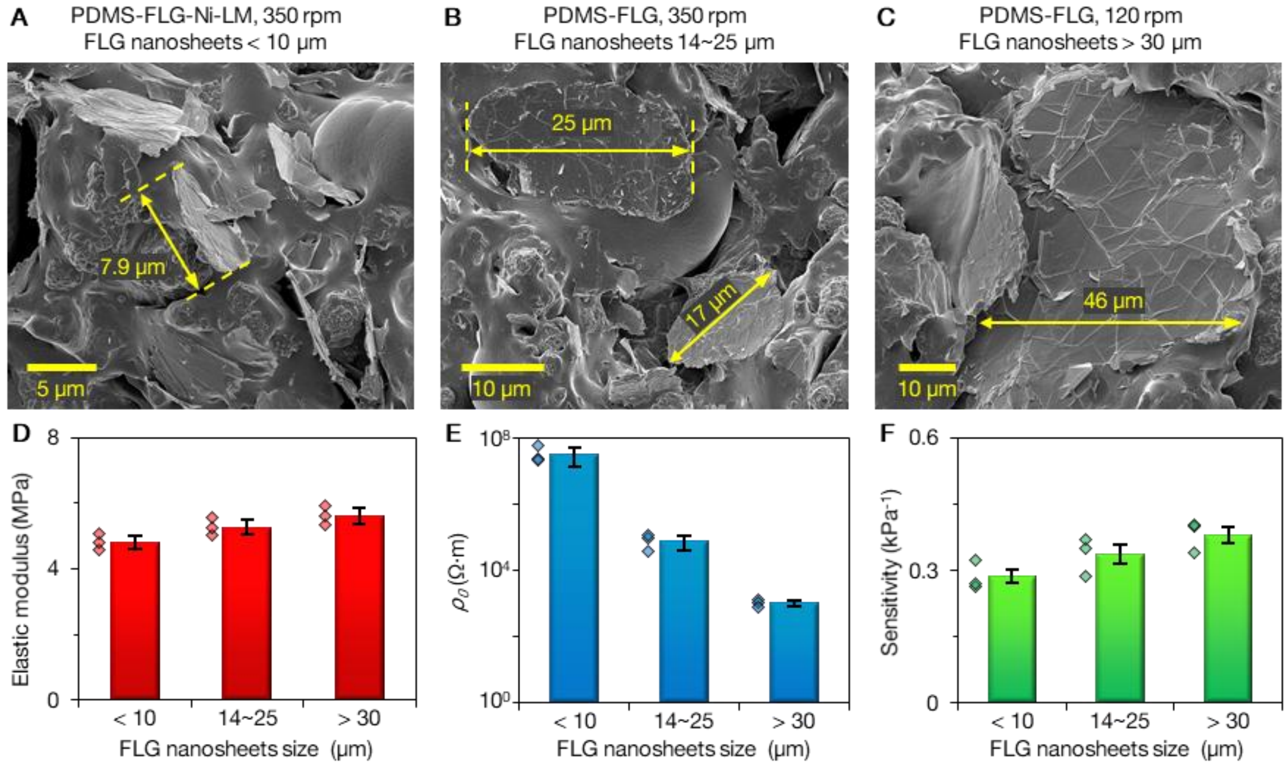

Supplementary Fig. 8.

(**A-C**) The SEM images of FLG nanosheets in composites prepared using different methods. Effects of FLG nanosheet sizes on the (**D**) elastic modulus, (**E**) initial resistivity, and (**F**) initial pressure sensitivity of APE. For all error bars in (**D** to **F**), n = 3 derived from different APE samples, data are presented as mean values +/- SD.

The diameter of FLG nanosheets used to prepare APE is usually above 30 μm and with a thickness of ~50 nm. Due to their high aspect ratio, FLG nanosheets are easily broken during stirring, especially under high shear forces. When FLG nanosheets was mixed with Ni particles, LM, and PDMS and stirred at a speed of 350 rpm, the high viscosity of the mixture and rapid stirring resulted in high shear forces, causing the vast majority of FLG nanosheets to be broken to less than 10 μm (Supplementary Fig. 8A). As a result, the broken FLG sheets cannot bridge the Ni particle chains well to build conductive paths, resulting in APE being almost insulating (resistivity > 30 MΩ·m) and a low pressure sensitivity of 0.287 kPa^{-1}.

To solve this problem, when preparing APE, we pre-dispersed FLG nanosheets in PDMS and mixed it with the Ni-LM-PDMS mixture at a low speed to maintain the large size of the FLG

nanosheets. As can be seen in Supplementary Fig. 8, B and C the lowest premixing speed can maintain the size of most FLG nanosheets above 30 μm, which significantly increases the conductivity of APE and enhances its pressure sensitivity by 33% to 0.382 kPa^{-1}.

Note that this potential breakage may only occur if a high-speed mixing process is used during composite preparation. We note that the FLG nanosheets maintain their structural integrity throughout subsequent compressive tests and under the sensor operational range.

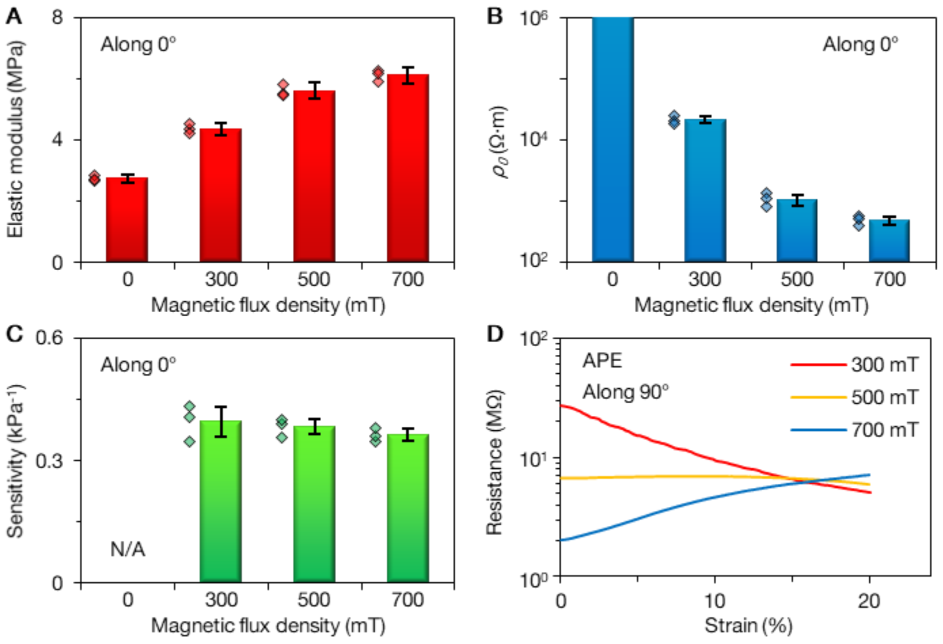

Supplementary Fig. 9.

Effects of the strength of curing magnetic field on the (**A**) elastic modulus, (**B**) initial resistivity, and (**C**) initial pressure sensitivity of APE. For all error bars in (**A** to **C**), n = 3 derived from different APE samples, data are presented as mean values +/- SD. (**D**) Resistance-compressive strain curves of APE samples cured in magnetic field with different strengths.

We investigate the influence of the curing magnetic field strength on the pressure sensing performance of APE, as shown in Supplementary Fig. 9. The isotropic sample cured without a magnetic field (strength of 0) is insulating (resistivity > 100 MΩ·m), demonstrating the necessity of the anisotropic particle network to enhance the conductivity of the sample. As the magnetic field strength increases, the elastic modulus and electrical conductivity of the obtained APE samples increase, yet their pressure sensitivity does not decrease significantly. The pressure sensitivity of APE samples cured in 300/500/700 mT magnetic fields differed by only 4%.

More importantly, compared with our previous research on aligned filler networks that only focused on the anisotropic sensitivity of composites(*44*), this work successfully eliminates the crosstalk interference caused by lateral stress deformation through the anisotropic structure design, demonstrating significant advancement. The magnetic field strength can adjust the anisotropy ratio of APE and the effect of lateral deformation (along 90°) on the sample resistance, which is crucial to

avoid interference caused by lateral strain (along 90°) when the sensor is working. When cured in a magnetic field of 500 mT, APE exhibits nearly constant resistance along the 90° direction (<3.5% variation within 17% strain). Even with 5% compression along 90°, the change in conductivity along 0° is less than 5%. As a comparison, when APE is compressed by 5% along 0°, its conductivity increases 2000 times, showing a change that is 4 orders of magnitude higher. This insensitivity to lateral deformation is important for APE sensors as it avoids signal interference caused by working on curved surfaces or being stretched.

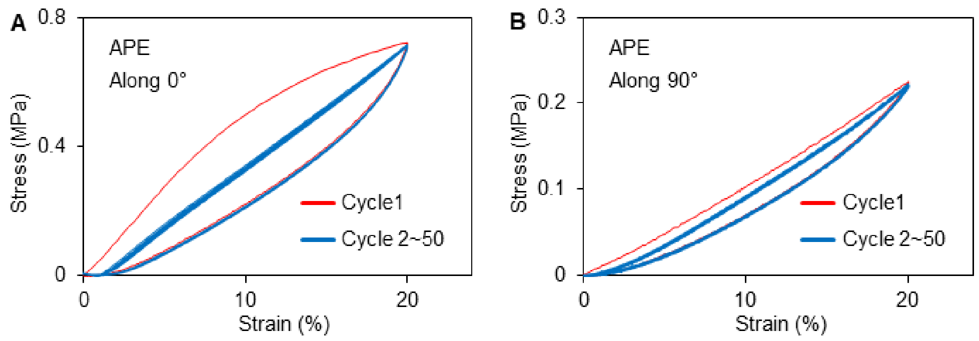

Supplementary Fig. 10.

The stress-compressive strain curves of APE along (**A**) 0° and (**B**) 90° under cyclic loading for 50 cycles.

In cyclic loading tests, APE demonstrates both irreversible and recoverable elastic hysteresis while maintaining mechanical stability in directions parallel (0°) and perpendicular (90°) to the Ni particle chain alignment. The first loading cycle exhibits pronounced elastic hysteresis, particularly along 0° where a stiffness softening phenomenon emerges at strains exceeding around 6%. This initial hysteresis arises from two concurrent mechanisms: irreversible particle rearrangement as closely packed Ni chains resist compression until critical strain triggers chain disintegration (irreversible elastic hysteresis), and recoverable viscoelastic dissipation in the polymer matrix (recoverable elastic hysteresis). After the second cycle, the particles in the composite redistribute and stabilise, eliminating the irreversible hysteresis, leaving only the inherent recoverable hysteresis of the elastomer. Subsequent cycles show basically overlapping curves, showing good mechanical stability (Supplementary Fig. 10).

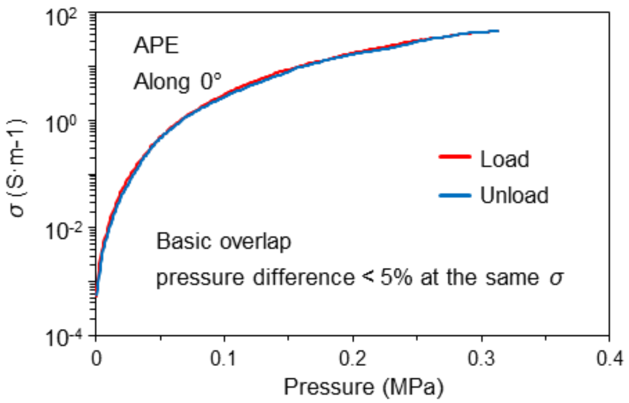

Supplementary Fig. 11.

The conductivity-pressure curves of APE during the loading and unloading phases.

While the composite exhibits intrinsic elastic hysteresis in stress-strain relationships (Supplementary Fig. 10), the conductivity-pressure response demonstrates negligible hysteresis due to stress-dominated conduction mechanisms. Regardless of whether the material is being loaded or unloaded, the same pressure always corresponds to the same conductivity, avoiding the impact of elastic hysteresis on pressure detection (Supplementary Fig. 11).

During each load-unload cycle, as the material undergoes loading, the conductive particles squeeze each other, resulting in higher stress and, therefore, lower particle contact resistance and higher overall conductivity. In the unloading phase, the material stress decreases faster due to elastic hysteresis, causing the conductivity to drop faster as well. In short, the conductivity of the composite primarily depends on the contact resistance between particles, which is largely determined by the stress rather than strain. This results in a negligible hysteresis in the conductivity-pressure relationship.

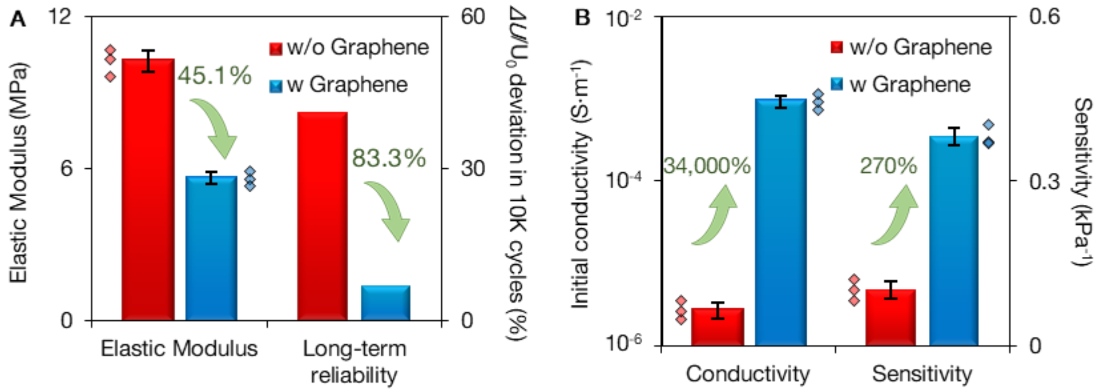

Supplementary Fig. 12.

The enhancing effect of graphene on the (**A**) flexibility and long-term stability, as well as (**B**) conductivity and sensitivity of composites. For composite materials without (w/o) graphene, a Ni/PDMS mass ratio of 1.6 is used to achieve optimal sensitivity of the composite. The introduction of graphene significantly reduces the need for Ni in composites. For all error bars, n = 3 derived from different composite samples, data are presented as mean values +/- SD.

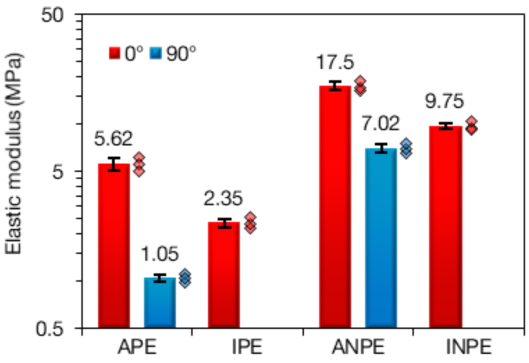

Supplementary Fig. 13.

The elastic modulus of APE, ANPE, and their isotropic versions. For all error bars, n = 3 derived from different composite samples, data are presented as mean values +/- SD.

Supplementary Fig. 13 compares the elastic modulus of APE and ANPE along 0° and 90°, as well as the modulus for isotropic porous (IPE) and non-porous (INPE) elastomers. It can be seen that the porous structure significantly reduces the elastic modulus of the composites along 0° by 68% (17.5 MPa for ANPE to 5.62 MPa for APE). As a result, the deformation of APE is > 3 times that of ANPE under the same pressure. More importantly, in non-porous ANPE, a large amount of stress and deformation during compression are shared by the PDMS matrix; while in porous APE, the porous structure between Ni particle chains cannot share the pressure, causing almost all stress to be concentrated on the Ni particle chains. These result in more pronounced filler network deformation and conductive particles contact, resulting in significant conductivity changes and extremely high sensitivity. Note that isotropic porous or non-porous composites (IPE and INPE) with identical filler content but not cured under an applied magnetic field are insulators.

In addition, APE also exhibits more significant stiffness anisotropy (anisotropic ratio of 5.4). This is because most of the deformation during compression along 90° is achieved through the deformation of 0-stiffness micropores, which greatly reduces the elastic modulus along 90° (1.05 MPa).

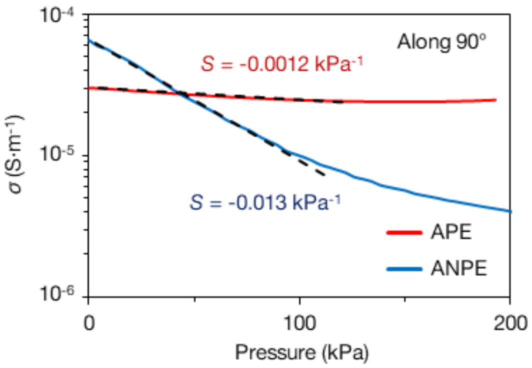

Supplementary Fig. 14.

The conductivity-pressure curves of APE and ANPE along 90°.

Supplementary Fig. 14 compares the conductivity-pressure curves of APE and ANPE along 90°. Along 0°, APE has a pressure sensitivity more than ten times higher than that of ANPE (fig. 3C). However, along 90°, APE shows a sensitivity of almost 0 (0.0012 kPa^{-1}), which is only 9% of that of ANPE (0.013 kPa^{-1}) (the principle is detailed in Supplementary Fig. 9). The sensitivity of APE along 0° is 320 times higher than that along 90°, which significantly reduces the interference of lateral deformation (along 90°) on the APE sensor.

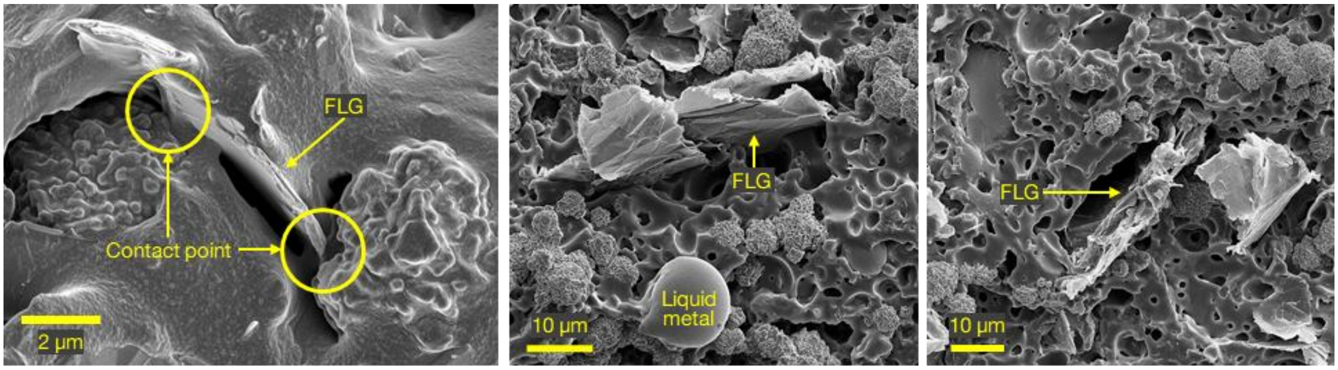

FLG nanosheets and LM droplets bridge Ni microparticles

Supplementary Fig. 15.

SEM images show FLG nanosheets and LM droplets bridging within and between Ni particle chains.

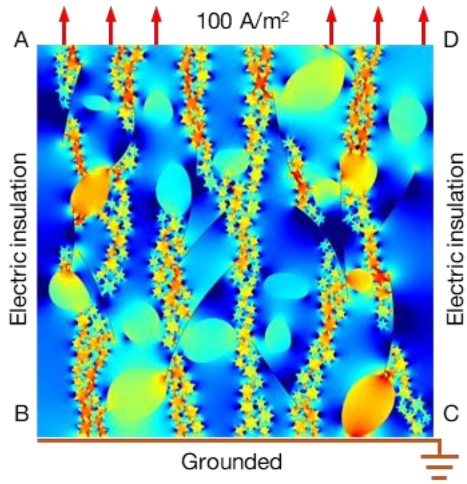

Supplementary Fig. 16.

The simulation settings for the electrical conductivity simulation.

We use COMSOL finite element software to simulate the resistivity of the APE and ANPE 2D models (100×100 μm) during a compression process. The thickness of the model is set at 1 μm. The models are established based on the SEM images. The sizes of the Ni (2~5 μm), pores (3~8 μm), FLG nanosheets (20~40 μm), and LM (15~30 μm) particles are equal to those in the actual composites. We use an electrical-mechanical coupled multi-field to simulate the mechanical deformation and resistivity of the model. The material parameters of the fillers and PDMS matrix (mainly including the elastic modulus, Poisson's ratio, conductivity, and dielectric constant) are consistent with the COMSOL material library. In the simulation model, we omit the gallium oxide layer on the surface of the EGaIn droplet. This is because the thickness of this gallium oxide film is only 1~3 nm(*S1*), which is much smaller than the diameter of LM droplets. Such a thin oxide layer does not affect the mechanical deformation of the LM droplets. Additionally, since the conductivity of both the LM droplet and the gallium oxide is much higher than that of the PDMS matrix, this extremely thin oxide layer also does not affect the simulation for the resistivity of the composite. The mechanical deformation and microstructure change of the model are simulated by applying a fixed displacement or pressure load to the upper side of

the model. Supplementary Fig. 16 shows the boundary conditions in the simulation of electrical conductivity. The left and right sides of the 2D model are electrically insulated, the lower side is grounded, and the current density through the upper side is fixed at 100 $A \cdot m^{-2}$. The resistivity of the model can be calculated based on the drop in electrical potential.

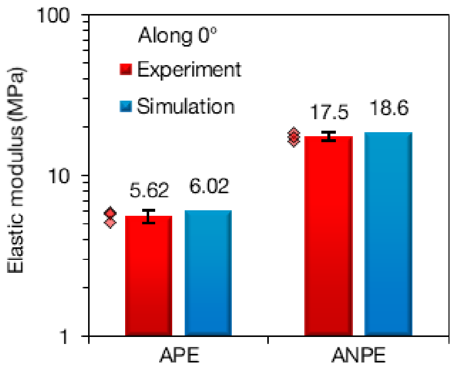

Supplementary Fig. 17.

The experimental and simulated elastic modulus of APE and ANPE. The simulated elastic modulus is calculated based on the simulated stress distribution and model deformation. For both error bars, n = 3 derived from different composite samples, data are presented as mean values +/- SD.

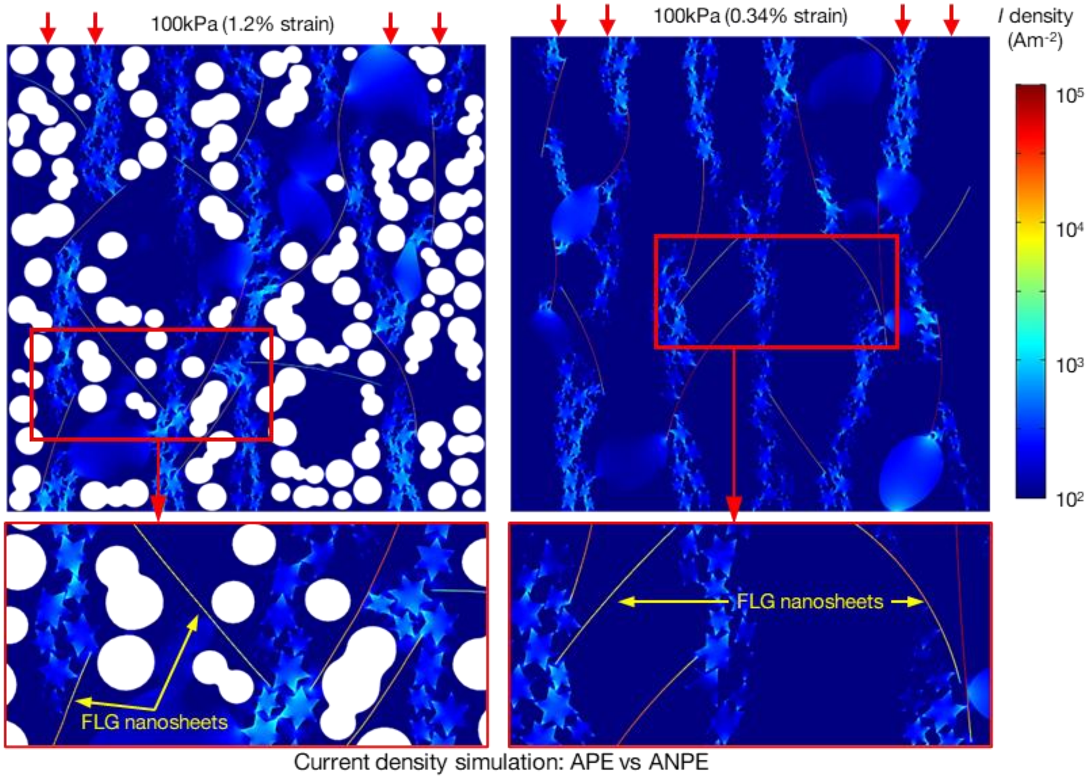

Supplementary Fig. 18.

Simulated current density of APE and ANPE models at 100 kPa pressure. The colour bar is offset to highlight the current flow in FLG nanosheets.

Due to the extremely high aspect ratio of FLG nanosheets, they are very thin and difficult to see in the 2D finite element model. Therefore, we enlarged the current density simulation images of APE and ANPE, and changed the colour bar value range of the current density (from 10~1000 Am^{-2} to 100~100,000 Am^{-2}) to highlight the high current density flowing through FLG nanosheets.

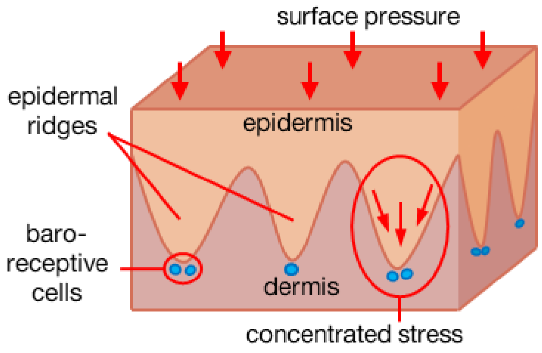

Supplementary Fig. 19.

The wavy structure of human skin.

In human finger skin, the rete pegs of the papillary layer on the uppermost dermis extend into the epidermis, forming a wavy interface. Under pressure, the pressure is concentrated on baroreceptive cells at the bottom of the wave structure, resulting in high pressure sensitivity of the skin(*45*). The surface structures of APE sensor is designed by imitating this natural structure.

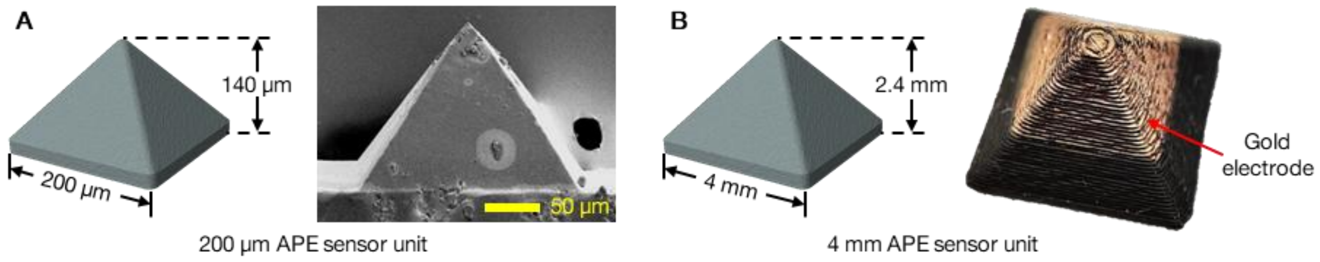

Supplementary Fig. 20.

The dimensions and optical photo of pyramid-shaped APE sensor units with side lengths of (**A**) 200 μm and (**B**) 4 mm. The top half of the sensor unit is evaporated with a layer of gold electrode for electrical testing (see Methods for details). For ease of experiments, unless otherwise specified, subsequent mechanical and electrical tests on a single sensor unit use the sensor unit with 4 mm side length.

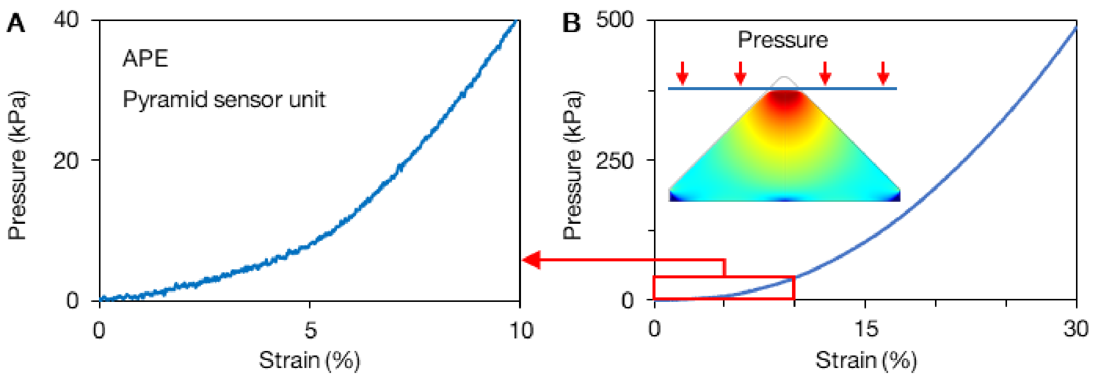

Supplementary Fig. 21.

The pressure-compressive strain curve of a millimetre-scale APE sensor unit.

At the beginning of compression, the sensor is only stressed at the tip with obvious stress concentration, which can produce large deformation and resistance changes under small pressure (Supplementary Fig. 21A). As the pressure increases, the contact area between the sensor tip and the contact surface increases significantly, resulting in a gradual increase in the equivalent compression modulus (Supplementary Fig. 21B).

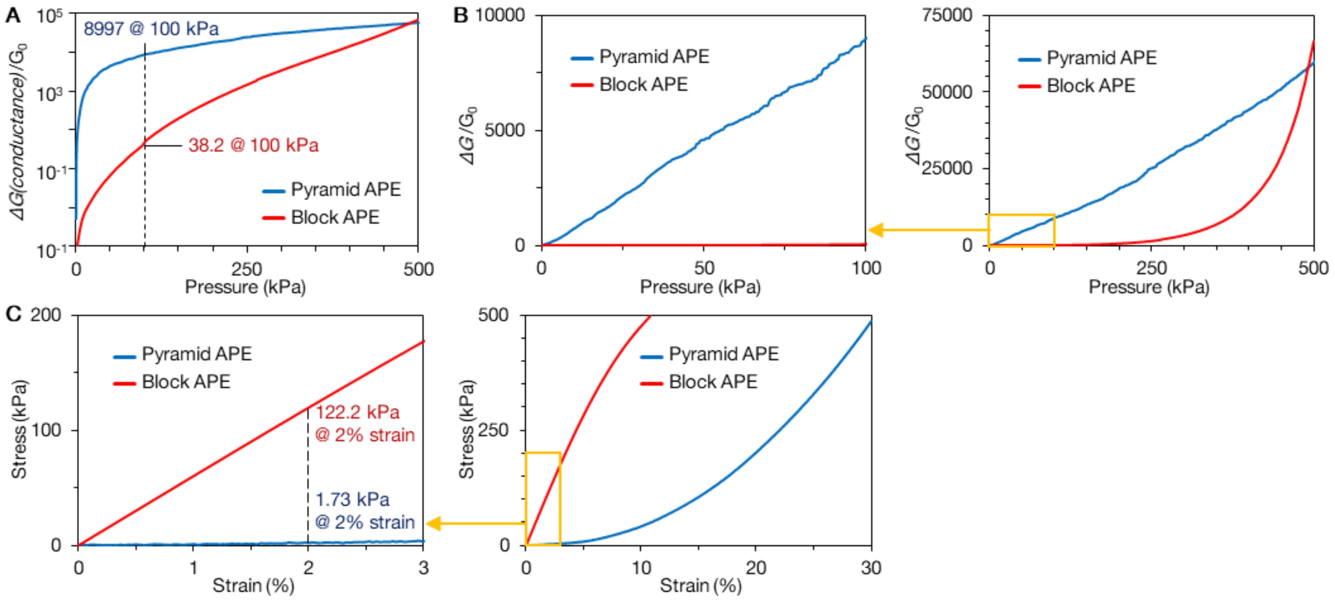

Supplementary Fig. 22.

Comparison of (**A-B**) relative conductance change ($\Delta G/G_0$)-pressure curves and (**C**) stress-strain curves between pyramid and block APE.

The pyramid-structured APE (Fig. 3C) exhibits sensitivities several hundred times higher than the block APE (Fig. 2C). This exceptional sensitivity originates from the linear conductance-pressure response enabled by the pyramid geometry, in contrast to the exponential behaviour of the block composite.

As shown in Supplementary Fig. 22, A and B, both the block and pyramid-structured composites show a conductance enhancement exceeding 50,000 times at 500 kPa, starting from the similar initial conductance. However, the block composite exhibits only minimal conductance change at low pressures due to its exponential conductance-pressure relationship, leading to a sensitivity of just 0.382 kPa^{-1} in the low-pressure region (a 38.2-fold conductance increase at 100 kPa). In contrast, the pyramid composite achieves a linear conductance increase of 8997-fold at 100 kPa (Supplementary Fig. 22B), corresponding to a sensitivity of 89.97 kPa^{-1}. This behaviour is primarily owing to the pyramid geometry, which undergoes significant deformation and conductance enhancement even under small pressures due to the stress concentration (Supplementary Fig. 22C). Consequently, the pyramid APE delivers highly linear and ultra-sensitive responses across the full 500 kPa range.

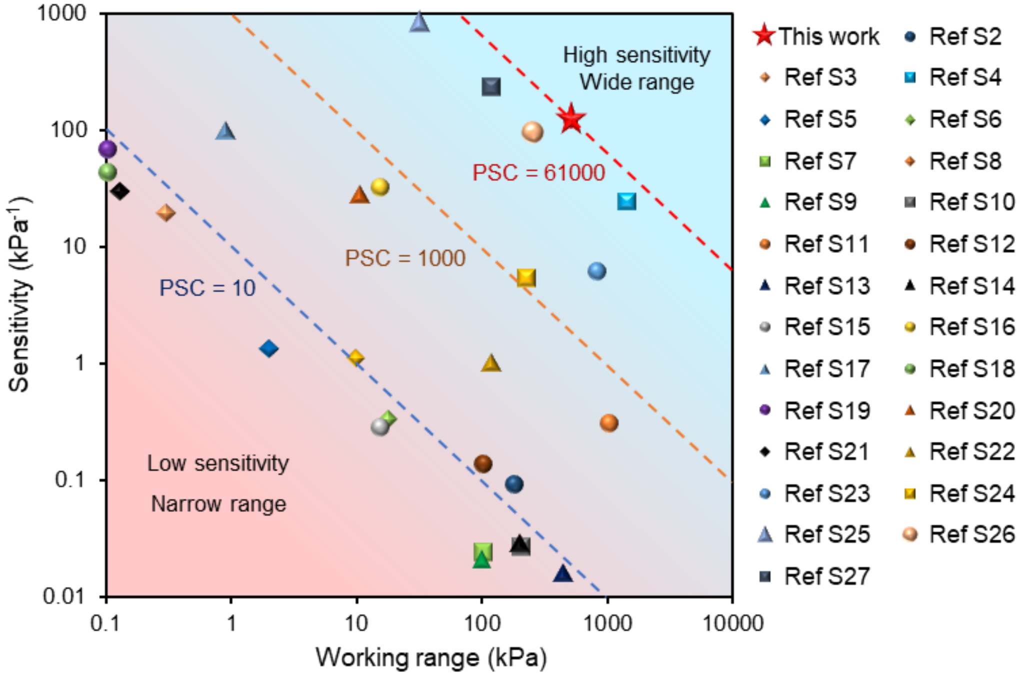

Supplementary Fig. 23.

Sensing performance comparison between the APE sensor and state-of-the-art flexible pressure sensors(*S2-S27*).

Compared with existing linear response flexible pressure sensors, APE demonstrates not only a high pressure sensitivity (122.7 kPa^{-1}) but also the largest pressure sensing coefficient (PSC, the product of pressure sensitivity and linear sensing range). The PSC of most flexible pressure sensors is around 10~1,000, with a few reaching 10,000. Combined with both high sensitivity and wide working range, APE exhibits an astonishing PSC of > 61,000, exceeding the state-of-the-art.

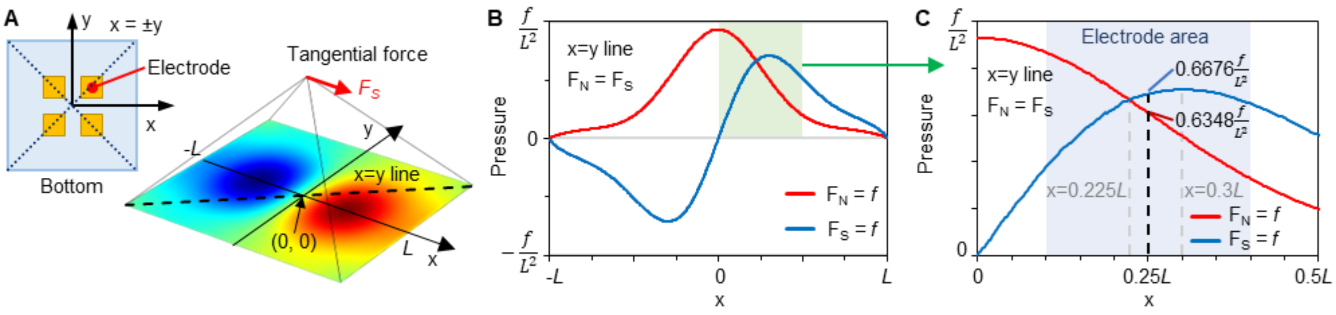

Supplementary Fig. 24.

(**A**) Simulated bottom pressure distribution of APE sensor unit under shear force and schematic diagram of electrode positions. (**B** and **C**) The bottom pressure distribution curves along x=y line of the APE sensor unit under normal or shear forces. The shaded region in C indicates the region covered by the electrodes.

The four bottom electrodes were positioned symmetrically on the two diagonals of the square bottom surface (x = ±y) on the sensor bottom (Supplementary Fig. 24A). Their positions are derived from simulations of the bottom pressure distribution on the x = y line under the normal and shear forces (Supplementary Fig. 24B). The design objective is to maximise the pressure sampled by each electrode for all three force components, while ensuring coverage of the maximum shear force-induced pressure zone and preventing any single force component from dominating the response.

Simulations in Supplementary Fig. 24C show that at x = ±0.225 L (denoted by the x = 0.225 L line), normal and shear forces of equal magnitude ($F_N = F_S = f$) generate nearly equal absolute bottom pressures. Furthermore, the electrode area should cover the region of maximum shear force-induced pressure (x ≈ ±0.3 L, denoted by the x = 0.3 L line in Supplementary Fig. 24C), enabling precise calculation of shear force from the pressure gradient. To balance these requirements, the four electrodes are placed at (x, y) = (±0.25 L, ±0.25 L), which yields simulated pressures of 0.6348 $\frac{F_N}{L^2}$ under normal force and ±0.6676 $\frac{F_S}{L^2}$ under shear force along the x-axis or y-axis, see Supplementary Fig. 24C. Each square electrode extends from ±0.1 L to ±0.4 L (side length of 0.3 L, see Supplementary Fig. 24C) to cover the high-pressure area. This optimised electrode configuration increases the signal-to-noise ratio and force resolution while enabling accurate force decoupling. Any measurement errors caused by minor positional deviations introduced during electrode fabrication can be corrected through calibration with standard loads, preserving reliable normal and shear force reconstruction.

For sensor unit with a side length of 4 mm (L = 2 mm), the four bottom electrodes are at (x, y) = (±0.5 mm, ±0.5 mm), where both normal and shear forces can produce large and relatively stable pressure. A normal force of 0.1 N can produce an equal pressure of $0.6348 \frac{0.1\ \mathrm{N}}{2\mathrm{mm}^2}$ = 15.87 kPa at these four points; while a shear force of 0.1 N (along the x-axis) can produce a pressure of $\pm 0.6676 \frac{0.1\ \mathrm{N}}{2\mathrm{mm}^2}$ = ±16.69 kPa at x = ±0.5 mm. Simulations of a sensor unit with a side length of 200 μm shows the same pattern. For this case, the coordinates of the four bottom electrodes of the microsensor unit are (x, y) = (±25 μm, ±25 μm).

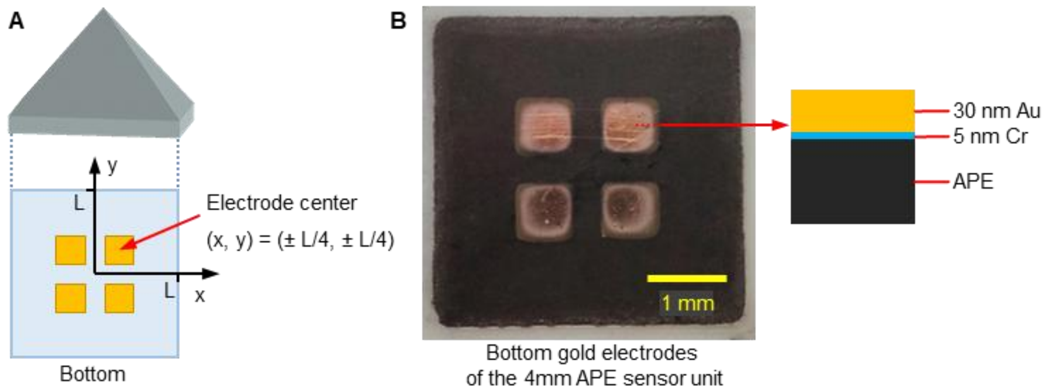

Supplementary Fig. 25.

(**A**) Location and (**B**) photo of the bottom electrodes of APE sensor unit with the side length of 4 mm.

The square-pyramid sensor employs a four-electrode base configuration to detect pressure distribution, where the averaged pressure correlates with normal force and the pressure gradient reflects the magnitude and direction of tangential force (see Fig. 3D to E, and detailed discussion in Section 4).

Although triangular pyramids could theoretically achieve force decoupling with three electrodes, their inherent error sensitivity severely limits practical reliability. This limitation arises because single-point measurement errors in triangular configurations propagate linearly into force calculations, introducing substantial deviations. By contrast, our four-electrode square-pyramid design leverages statistical oversampling, reducing random error variance by 25% ($\sigma^2/4$ vs. $\sigma^2/3$ for triangular systems) through redundancy-weighted averaging. Furthermore, the symmetrical four-electrode arrangement allows residual-based outlier detection, which is absent in three-electrode systems with insufficient degrees of freedom.

While pentagonal/hexagonal pyramids with additional electrodes could marginally enhance stability, their nonlinear benefit scaling renders them impractical. Specifically, using complex geometric shapes and increasing electrode count compromises manufacturing feasibility and amplifies computational complexity in solving high-dimensional force-decoupling equations. We therefore adopt

the square-pyramid configuration as the optimal compromise, achieving robust force discrimination with minimal electrodes while maintaining fabrication and operational simplicity.

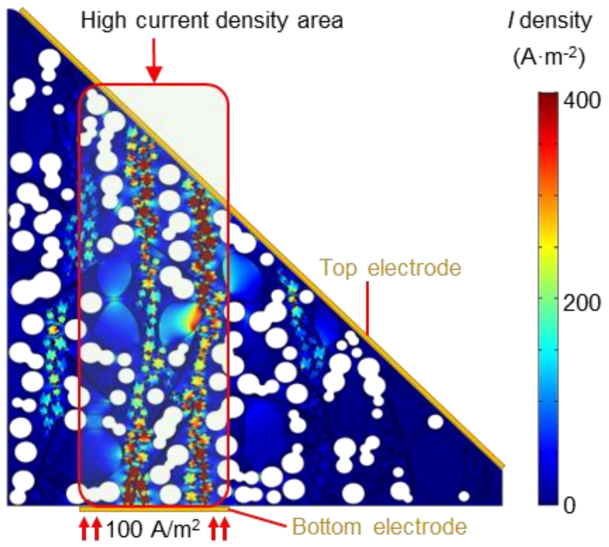

Supplementary Fig. 26.

Current density simulation shows high current density area in the pyramid sensor.

The pyramidal-structured sensor exhibits an uncomplicated electrical potential distribution, primarily attributed to the confined geometry of the bottom electrodes and the composite material's significant anisotropic conductivity characteristics. As illustrated in Fig. 2A, the composite demonstrates markedly enhanced conductivity along the alignment direction (vertical direction of the pyramid sensor) compared to that perpendicular to the alignment direction (horizontal direction of the sensor). This anisotropic behaviour enables the sensor to be regarded as an aggregation of longitudinally aligned strip regions connected in parallel via a common top electrode, with only four strips selectively activated through the bottom electrode array.

Notably, the non-planar topology of the top electrode exerts negligible influence on the potential distribution within the sensor, as evidenced by the current density simulation in Supplementary Fig. 26. The output signals of the four bottom electrodes mainly depend on the strength of the conductive pathways within their respective longitudinal regions between them and the common electrode (high current density area in Supplementary Fig. 26), that is, the pressure in this region, and are basically

independent of the adjacent regions. This allows the sensor to basically preserve the intrinsic linear force response of the composite material, even with the non-planar top electrode.

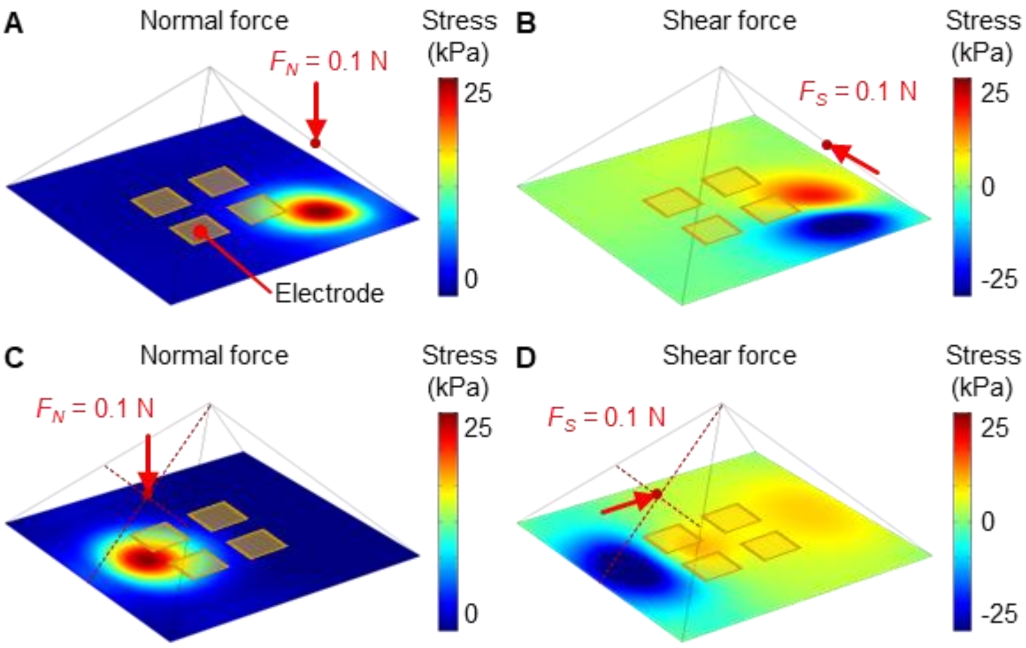

Supplementary Fig. 27.

Simulated bottom pressure distribution of the 4 mm APE sensor unit under (**A**) normal force and (**B**) shear force applied at the midpoint of the oblique edge, and (**C**) normal force and (**D**) shear force applied at the centre point of the side of sensor unit. The golden squares at the bottom indicate the positions of the electrodes.

The location of the applied force affects the pressure distribution at the sensor base. When force is applied at the sensor tip, the bottom pressure field exhibits a symmetric pattern (even function from normal force plus odd function from shear force, symmetric about the centre). By contrast, when force is applied at the side of the sensor unit, the bottom pressure shifts and becomes asymmetric, as shown by the finite-element simulations inSupplementary Fig. 27. In this case, the existing 4-electrode configuration is insufficient to determine the position of the force applied.

In theory, such shifts in pressure distribution could be detected by increasing the electrode density, such as adding three additional electrodes at the centre and along the x- and y-axes. This approach can provide sufficient data (7 electrodes for 7 degree-of-freedom) for reconstructing the magnitude, direction, and position of the applied force (3D force and 2D position with total 5 degree-of-freedom). However, implementing this approach would require substantial optimisation of high-precision

manufacturing processes, computational models, and calibration algorithms, which is beyond the scope of our current work.

To address this issue in practice, we adopted a miniaturised sensor array architecture (detailed in the section about the APE microsensor array). Each unit in the array measures only 200 µm, with an effective spatial resolution of 300 µm. Since most objects relevant to robotic manipulation or daily activities exceed 1 mm in size, the majority of contact events occur on the sensor tips rather than the sidewalls, thus minimising lateral contact interference. This strategy enables the array to reconstruct contact positions and force directions with high spatial resolution without the need for complex per-unit electrode layouts.

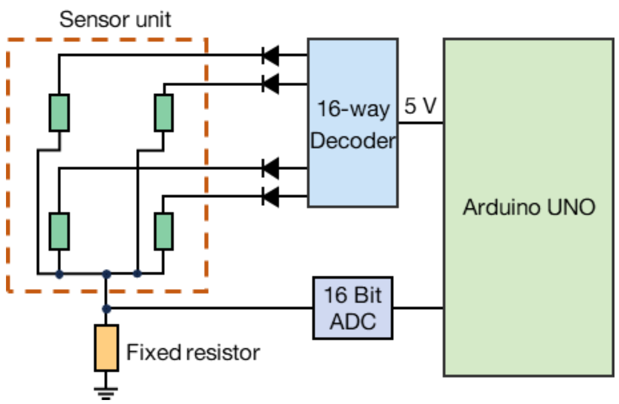

Supplementary Fig. 28.

The schematic diagram of the pressure measurement circuit.

Supplementary Fig. 28 shows a pressure measurement circuit based on an APE sensor unit controlled by an Arduino UNO. First, the high potential (5V) of the Arduino is connected to the four bottom electrodes of the sensor through a 16-way Decoder. The output potentials of different channels of the Decoder are switched by the Arduino program to traverse the four electrodes (frequency of 100 Hz). A diode is connected in series between the Decoder and each bottom electrode to prevent the remaining three electrodes from interfering with the measurement. The top electrode of the sensor unit is connected in series with a fixed resistor and then connected to ground. The divided voltage on the fixed resistor is read by Arduino through a 16-bit analog-to-digital converter (16-bit ADC) as the output signal of the APE sensor. As pressure increases, the sensor resistance decreases, causing the potentials of four bottom electrodes to rise.

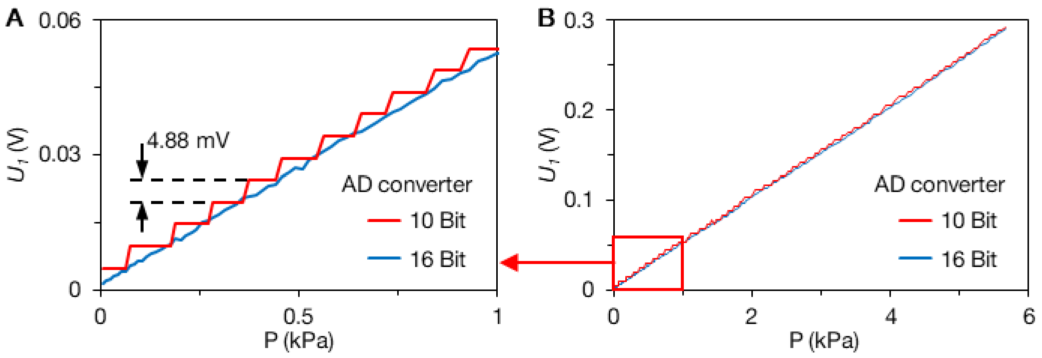

Supplementary Fig. 29.

Potential-pressure curves of the 1st electrode of the sensor unit in circuit with/without a 16-bit analog-to-digital converter at (**A**) 0-1 kPa and (**B**) 0-6 kPa.

Without the 16-bit ADC, the reading resolution of Arduino UNO is only 10 bit. In this case, the voltage resolution of the circuit is 4.88 mV, corresponding to a poor pressure resolution of 0.09 kPa. After using the 16-bit ADC, the theoretical voltage resolution of the measurement circuit is increased 64 times to 0.0763 mV, with an actual pressure resolution of < 0.01 kPa.

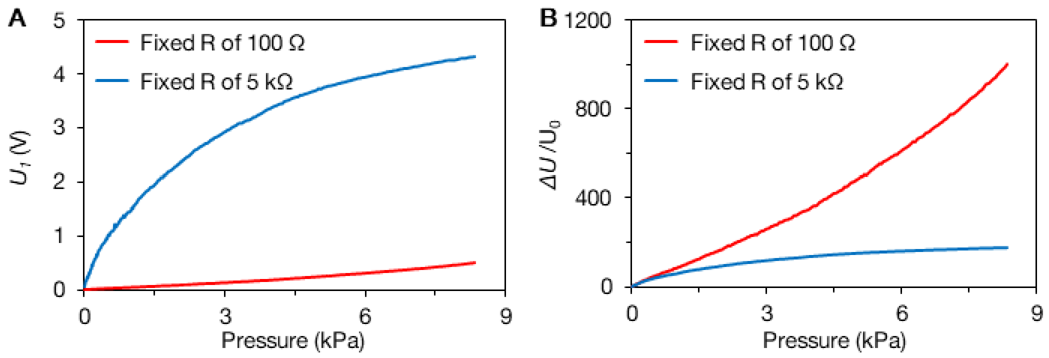

Supplementary Fig. 30.

(**A**) Potential-pressure curves and (**B**) Relative potential change-pressure curves of the 1st electrode of the sensor unit in circuit with a fixed resistor of 100 Ω and 5 kΩ.

The resistance of the fixed resistor in measurement circuit will significantly affect the sensitivity and detection range of the APE sensor. For a sensor with an initial resistance of about 1 MΩ, using a fixed resistor of 100Ω results in an initial voltage signal of ~0.5 mV (Supplementary Fig. 30A). The output voltage can rise linearly within a wide pressure range with a high sensitivity (Supplementary Fig. 30B). When using a fixed resistor of 5 kΩ, the initial voltage signal of the sensor increases to 25 mV, and the output voltage rises rapidly as the pressure increases and tends to saturation after exceeding 2 V (Supplementary Fig. 30A), resulting in nonlinear response and low sensitivity (Supplementary Fig. 30B).

In summary, with the resistance of fixed resistor set to 0.01% of that of APE sensor, a low initial voltage signal of approximately 0.5 mV provides the sensor with not only a linear voltage-pressure response but also a wide sensing range

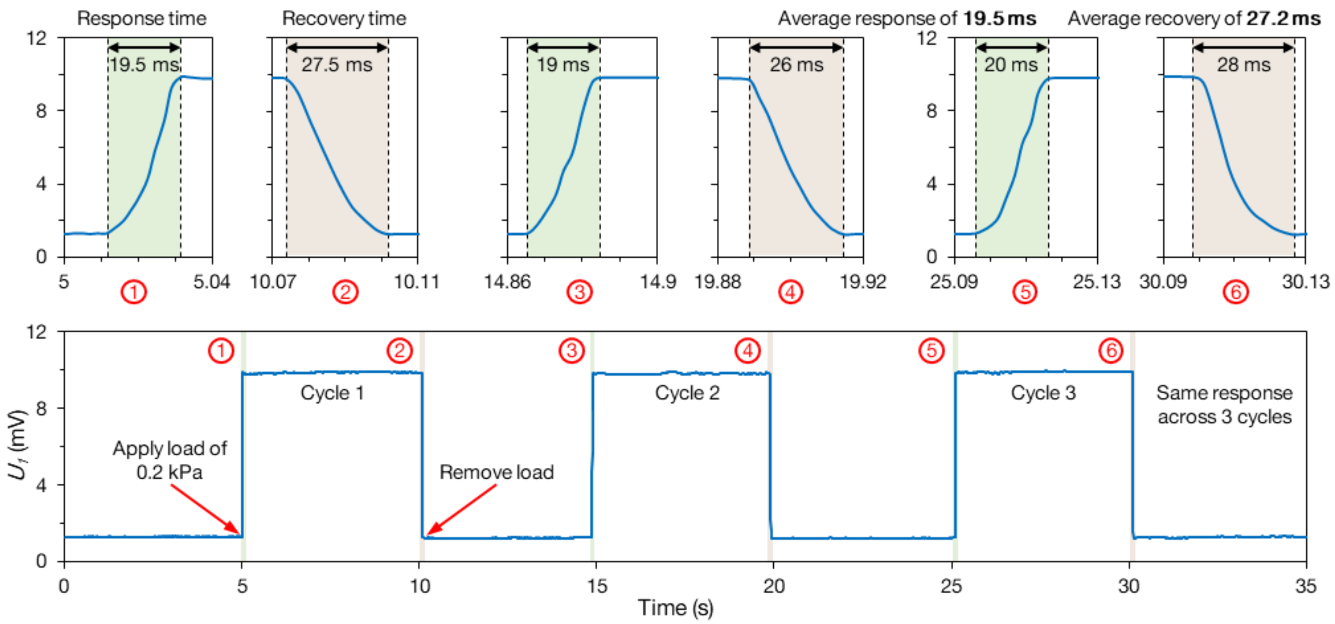

Supplementary Fig. 31.

Response and recovery time of the sensor unit under a 0.2 kPa square wave load for 3 cycles.

To quantify the response speed, the sensor unit was vertically loaded and unloaded at a constant pressure of 0.2 kPa for three cycles. The potential-time curve of the bottom electrode, along with the magnified view of all response and recovery regions (Supplementary Fig. 31), reveals average response (0% to 100%) and recovery (100% to 0%) times of 19.5 ms and 27.2 ms, respectively. These values are comparable to the ~30 ms response time of human skin(*50*), demonstrating that our sensor achieves rapid response with minimal latency.

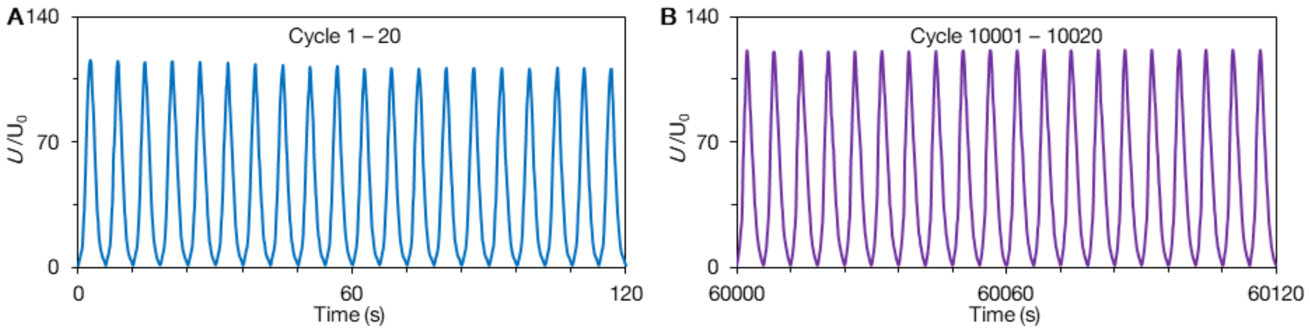

Supplementary Fig. 32.

Relative potential-time curves of the 1st electrode of the sensor unit in the (**A**) first 20 cycles and (**B**) cycles 10,001-10,020 of 12,000 cycles of a 0-1.6 kPa cyclic loading test.

In the early loading cycles of a newly fabricated sensor, the particle distribution within the composite material has not yet stabilised, resulting in non-recoverable elastic hysteresis, which manifests as an increase in the sensor's static resistance and a gradual decrease in the output voltage during the initial cycles of the test. However, after warm-up cycles, the particle distribution within the composite stabilises, and the response curves in subsequent cycles largely coincide. To confirm this, we have conducted a 12,000-cycle compression test on the sensor at 1.6 kPa (Fig. 3I). It can be observed that the sensor's output signal stabilises after the 400th cycles, demonstrating its long-term stability.

More importantly, as shown in Supplementary Fig. 32, the relative voltage-pressure curve of the sensor for each cycle remains consistent. The ratio of its output potential at 1.6 kPa to that at 0 kPa remains basically unchanged. Specifically, Supplementary Fig. 32A plots the output voltage during cycles 1-20 as a ratio to the initial voltage at cycle 1, whereas Supplementary Fig. 32B shows the ratio for cycles 10,001-10,020 relative to the initial voltage at cycle 10,001. Although the output voltage gradually decreases during the first ~400 cycles, the relative response to pressure remains largely constant throughout 10,000 cycles. For example, at 1.6 kPa and 0 kPa, the voltages were 105.63 mV vs 0.92 mV (ratio 114.8) in cycle 1, and 54.32 mV vs 0.46 mV (ratio 118.1) in cycle 10,001. This confirms that the sensor's force response and sensitivity remain stable over 10,000 cycles.

In practical use, the sensor will be calibrated based on its static resistance and output voltage at 0 kPa as the initial potential before measurement, and calculate the force based on the relative voltage change, ensuring consistent result under the same load. Therefore, differences in static resistance will

not cause measurement discrepancies. The relative potential-pressure curve of the sensor during the 12,000 cycles is almost unchanged with a stable sensitivity of ~70 kPa^{-1}, reflecting a stable sensing performance.

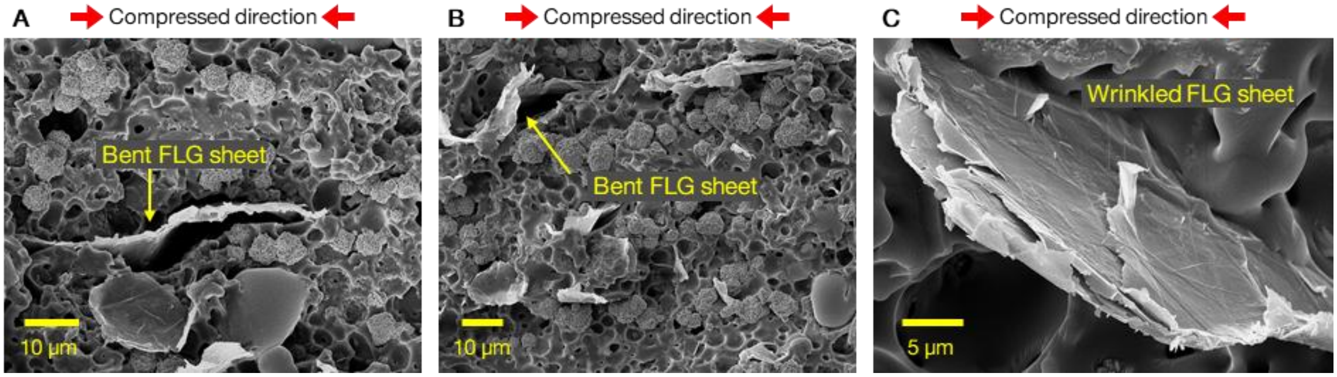

Supplementary Fig. 33.

SEM images showing (**A** and **B**) bent and (**C**) wrinkled FLG nanosheets in APE sensors after 100 cycles of loading at 1 MPa (compression direction is perpendicular to the sensor bottom).

The measurement range of the pyramid-shaped APE sensor is determined by its linear response range. Beyond this range, the pyramidal structure undergoes appreciable geometric nonlinearity: the pyramid will expand laterally, causing lateral deformation of the sidewalls and generating shear forces at the bottom. This effect produces a nonlinear coupling between normal and shear forces at the electrode interface. Under such conditions, Equation (3) which is derived under small deformation assumptions no longer provides accurate results. Accordingly, we define 500 kPa (the linear range shown in Fig. 3C) as the upper detection limit.

We note that the FLG nanosheets are vulnerable only if excessive shear during high-speed mixing is used in composite preparation. To avoid this, we use a low mixing speed of 120 rpm (detailed in Supplementary Fig. 8). Once cured, the inherent flexibility of FLG nanosheets protects them from operational damage. As evidence, Supplementary Fig. 33 presents SEM images after 100 cycles of 1 MPa triangular-wave loading (10 s per cycle), revealing only bending and surface wrinkling with minimal rupture of some FLG sheets. A pressure of 500 kPa is therefore far below the threshold that could compromise the sensor or the FLG sheets. These results confirm that within the linear range, the sensor operates safely without risk of FLG sheet failure.

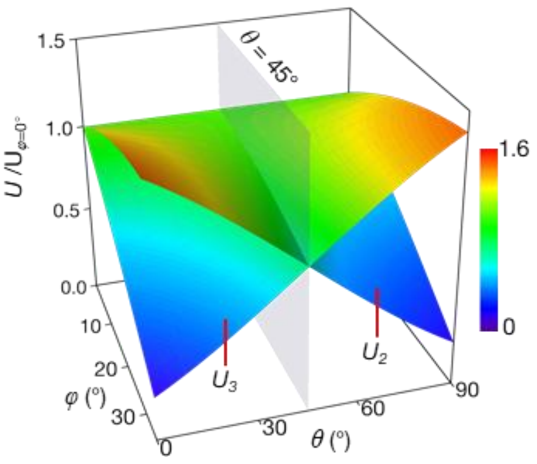

Supplementary Fig. 34.

Simulated relative potential of bottom electrodes 2 and 3 of APE sensor unit under different force directions. Since the positions of electrodes 2 and 3 are symmetric about θ = 45°, their simulated relative potentials are also symmetric about θ = 45°.

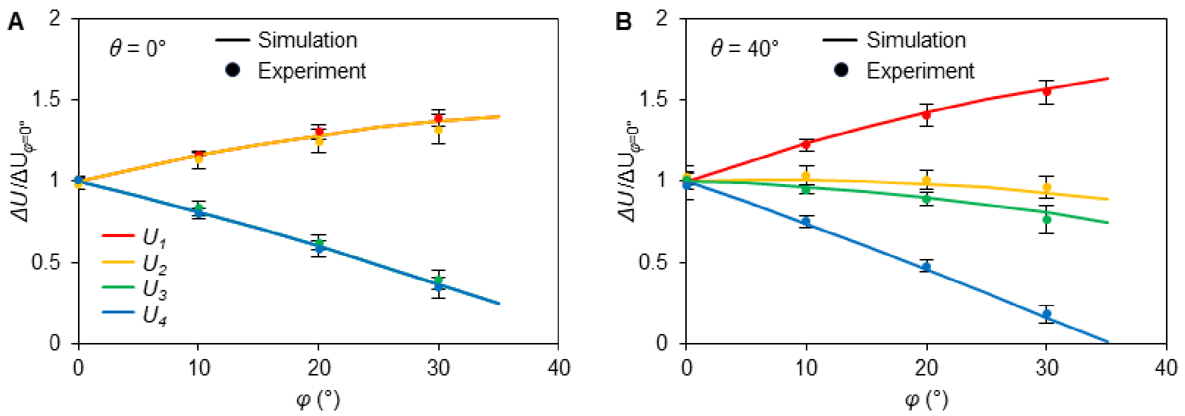

Supplementary Fig. 35.

Comparison of simulation and experimental results of the relative potential *vs* φ angle curves of the four bottom electrodes when the shear force direction θ is fixed at (**A**) 0° and (**B**) 40°. For all error bars, n = 5 derived from different APE sensor units, data are presented as mean values +/- SD.

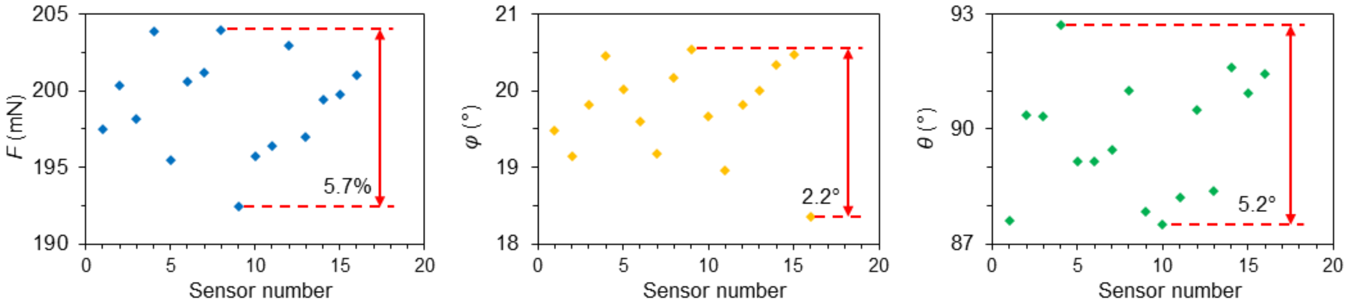

Supplementary Fig. 36.

The force, φ and θ measured by different batches of sensors under the same load.

To verify the consistency across devices, we standardised tests on 16 sensors from 4 batches, including both force response and force angle recognition. As stated in response to the previous comment, each device was automatically calibrated before testing to eliminate the influence of static resistance. The results show that under the same load of 0.2 N ($\varphi = 20°$, $\theta = 90°$), the maximum differences of force, φ and θ measured by different sensors are 5.7%, 2.2°, and 5.2°, respectively. This demonstrates good repeatability across devices.

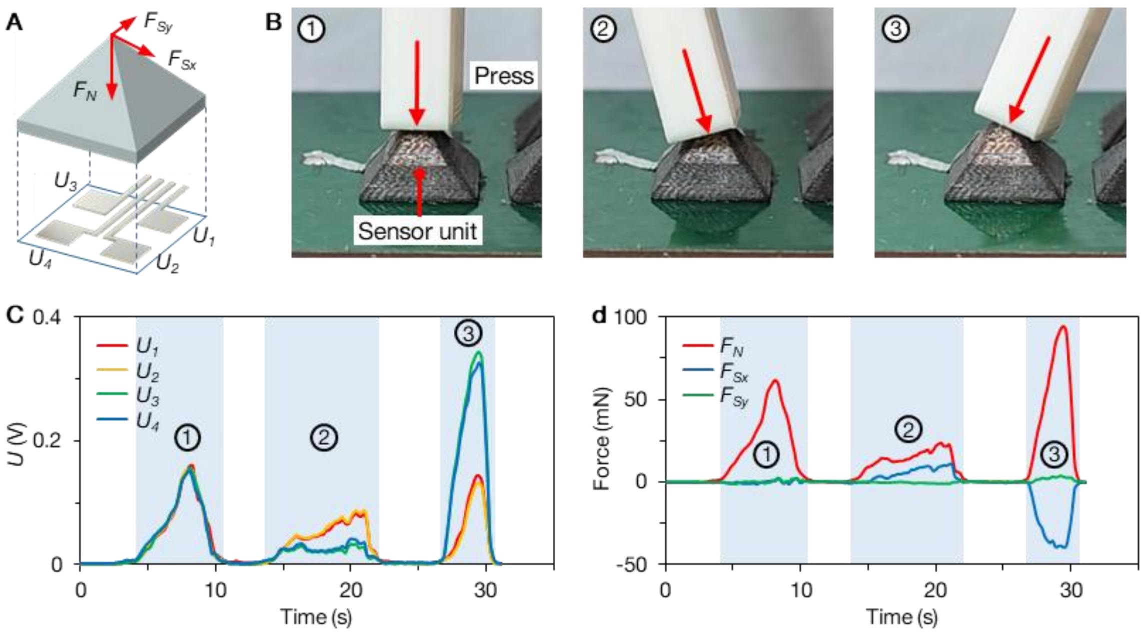

Supplementary Fig. 37.

Real-time 3D force sensing capability of a sensor unit. (**A**) Decomposition of the oblique force in the spherical coordinate system and potentials on the four bottom electrodes. (**B**) Photos of the 4 mm APE sensor unit when pressed in different directions. (**C**) Potential-time curves and (**D**) calculated forces-time curves of APE sensor unit during 3D force sensing test.

The force on the sensor can be divided into normal force (F_N) along the z axis and shear forces along the x-axis (F_{Sx}) and y-axis (F_{Sy}) (Supplementary Fig. 37A). We continuously applied three forces with different magnitudes and directions on a 4 mm sensor unit to demonstrate its real-time 3D force sensing capability. The three forces are the normal force, the oblique force to the right and the oblique force to the left, as shown in Supplementary Fig. 37B. It can be seen from Supplementary Fig. 37C that the signals U_1 ~ U_4 of the four bottom electrodes of the sensor basically overlap under normal force, but there are differences under oblique force. The three-component force-time curves calculated in real time based on the electrode potential-time curves are shown in Supplementary Fig. 37D. Supplementary Video 1 visually demonstrates the real-time calculation of force magnitude and direction.

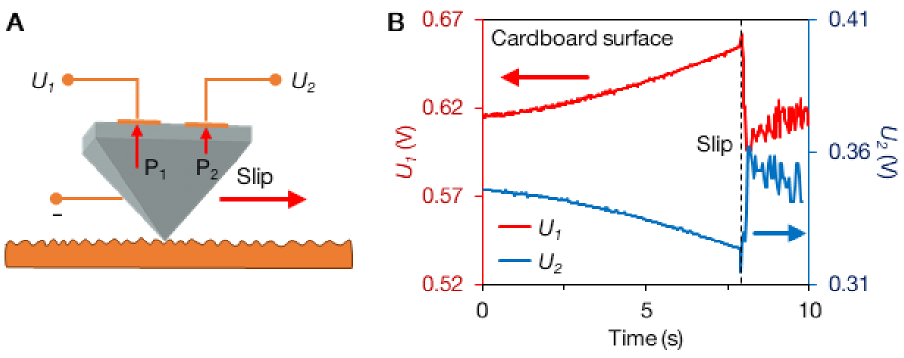

Supplementary Fig. 38.

(**A**) Schematic diagram of the APE sensor unit sliding on the cardboard surface. (**B**) The potential-time curves of the electrodes on the front and rear sides along the sliding direction.

When the sensor unit slides to the right on the contact surface (Supplementary Fig. 38A), the pressure on the left electrodes (P1 and P3) will momentarily decrease while the pressure on the right electrodes (P2 and P4, see electrode positions in Fig. 4a) will increase simultaneously due to the sudden drop in friction. This causes potential jumps in opposite directions but similar amplitudes on the left and right electrodes (see the potential changes on the P1 and P2 electrodes shown in Supplementary Fig. 38B). This can avoid the misjudgement of sliding caused by a sudden reduction in normal force.

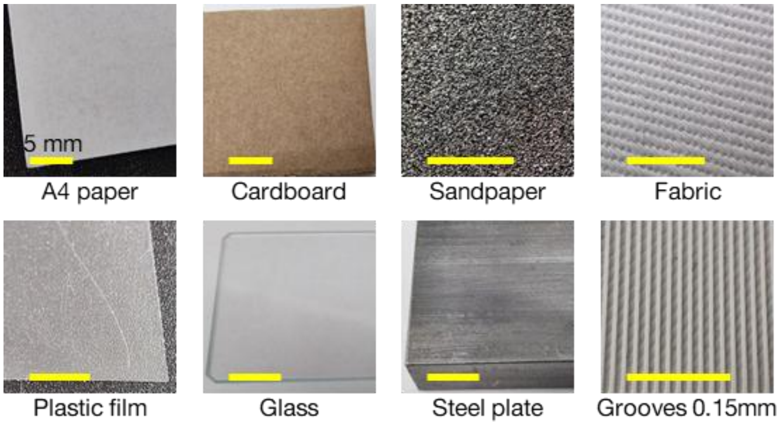

Supplementary Fig. 39.

Photos of different substrate materials. Scale bars are 5 mm.

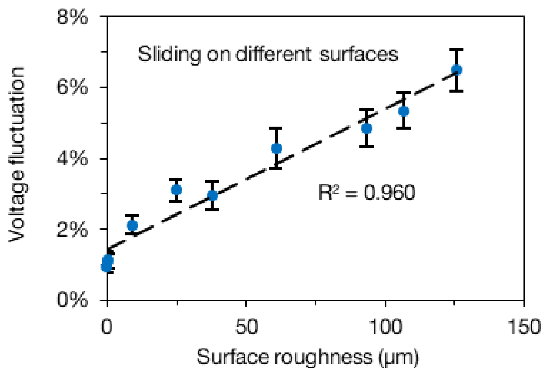

Supplementary Fig. 40.

Voltage fluctuations of the APE sensor when sliding on 10 substrates with different surface roughness. The linear correlation R^2 between voltage fluctuation and surface roughness is 0.96. The 10 substrates and their roughness are: Glass (0.007 μm), Plastic (0.17 μm), Steel (0.33 μm), A4 paper (9.1 μm), Cardboard (24.7 μm), Tissue (37.8 μm), Sandpaper (60.7 μm), 3D-printed grooves 0.1 mm (93.3 μm), Fabric (106.7 μm), 3D-printed grooves 0.15 mm (126 μm). Surface roughness is measured by a Bruker Dektak XT Stylus Profilometer.

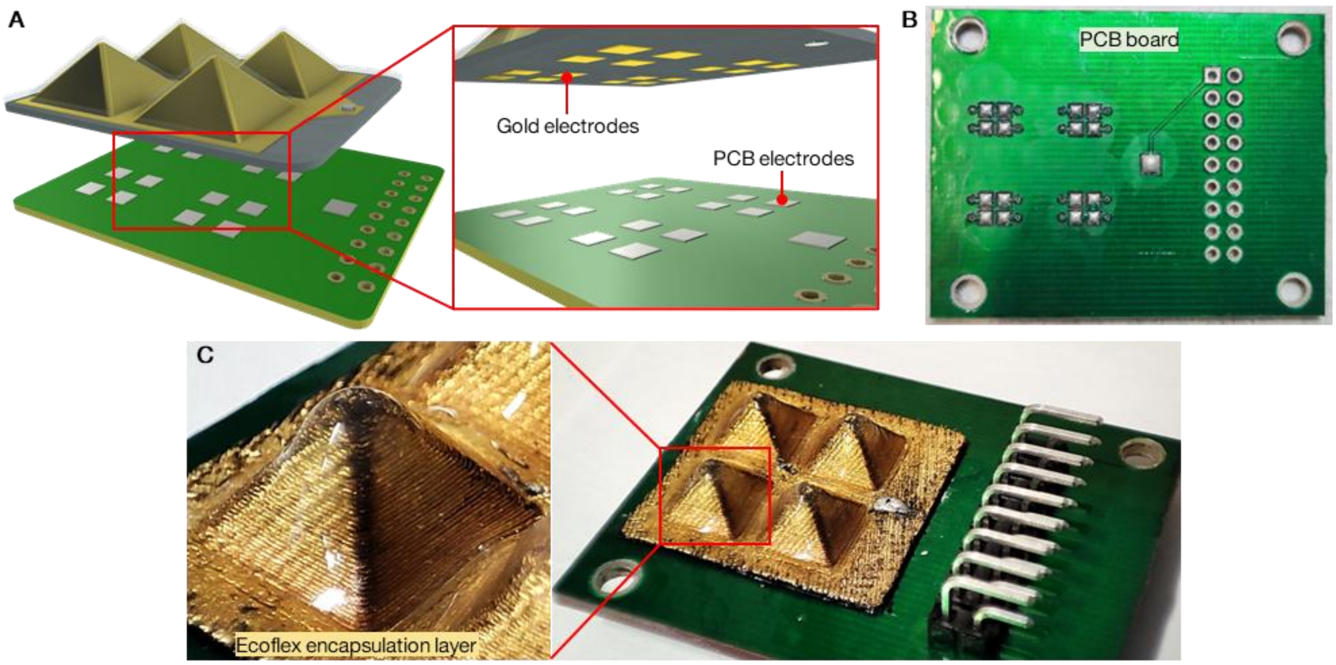

Supplementary Fig. 41.

Schematic of the assembly of the sensor array with the designed PCB. (**A**) The schematic shows the bottom electrodes of the sensor array corresponding to the electrode array on the PCB. (**B**) Photograph of the designed PCB. (**C**) Photograph of the APE force sensor array soldered on PCB board. Inset shows the Ecoflex encapsulation layer on the sensor array.

The APE sensor array shown in Supplementary Fig. 41A comprises of 4 sensor units. The sensor array features a top layer of evaporated gold electrode and 16 bottom electrodes (4 per unit). It is soldered on a designed PCB with a low melting point alloy (see Methods for details), and encapsulated with an Ecoflex layer on top to protect the electrodes from wear and tear (see the inset in Supplementary Fig. 41C).

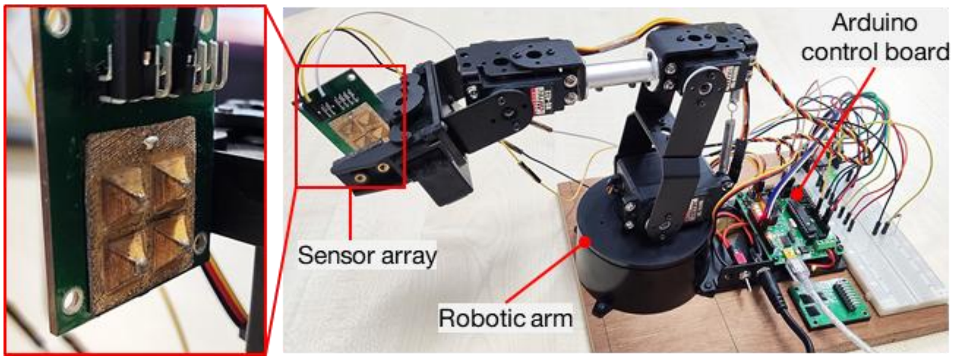

Supplementary Fig. 42.

Photograph of the APE sensor array mounted on the gripper of a robot manipulator. The voltage signals of the sensor array read by Arduino control the movement of the manipulator in real time.

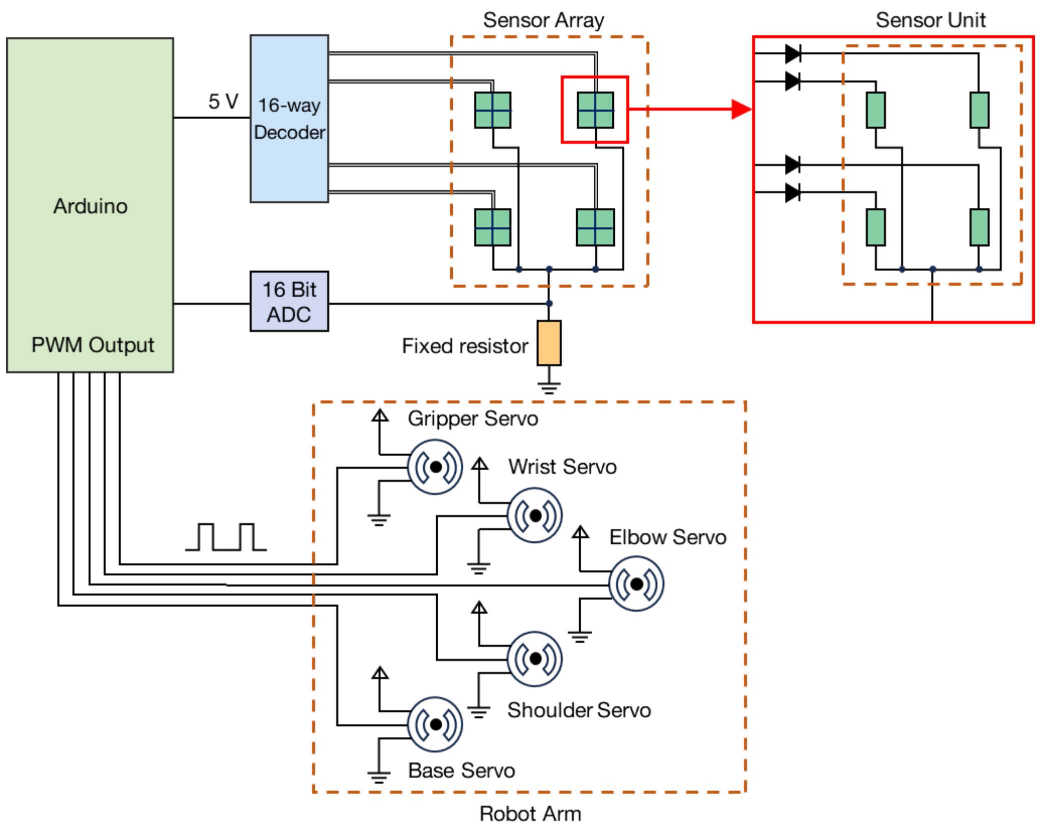

Supplementary Fig. 43.

The schematic diagram of the pressure measurement and feedback-control circuit of the robotic arm equipped with APE sensor array.

Supplementary Fig. 43 shows the real-time control circuit of the robotic arm equipped with APE sensor array controlled by an Arduino board. First, the high potential (5V) of the Arduino is connected to the 16 bottom electrodes (4 sensing units) of the sensor array through a 16-way Decoder. The output potentials of different channels of the Decoder are switched by the Arduino program, traversing 16 electrodes (frequency of 250 Hz). A diode is connected in series between the decoder and each bottom electrode to prevent the other electrodes from interfering with the measurement. The top electrode of the sensor array is connected in series with a fixed resistor and then connected to ground. The divided voltage on the fixed resistor is read by Arduino through a 16-bit ADC as control signals for the robotic arm. Arduino calculates force magnitude and direction in real time through pre-written programs, and controls the movement of 5 pre-programmed servos according to set trigger conditions, such as reaching the pressure threshold and the occurrence of sliding.

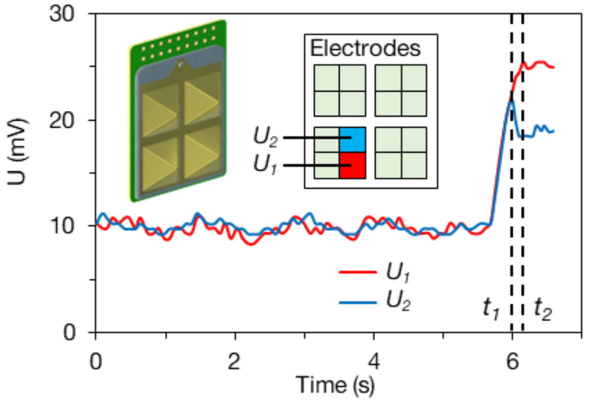

Supplementary Fig. 44.

Voltage signal-time curves of two representative electrodes of the APE sensor array during the process of the robotic arm clamping an A4 paper tube. The inset shows the location of two selected electrodes. The t_1 and t_2 correspond to t_1 and t_2 in Fig. 5C, meaning the paper tube is clamped (t_1) and lifted (t_2).

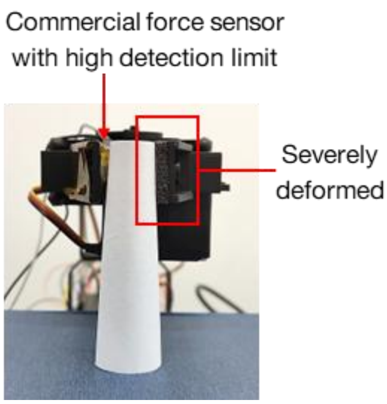

Supplementary Fig. 45.

Photos of the paper tube being clamped by a manipulator equipped with a commercial force sensor.

The manipulator can damage A4 paper tube due to the high detection limit of commercial force sensor.

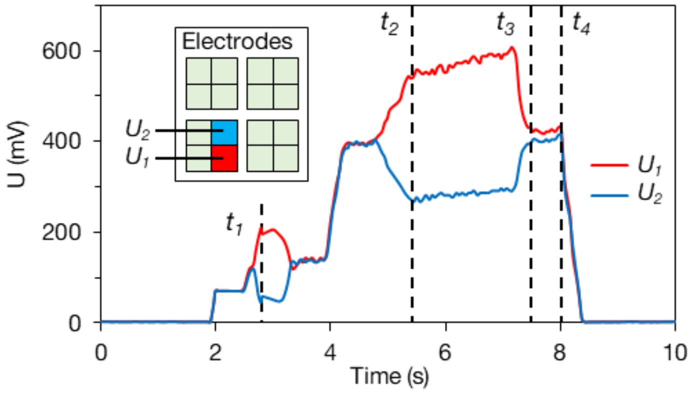

Supplementary Fig. 46.

Voltage signal-time curves of two representative electrodes of the APE sensor array during the process of the robotic arm transferring a steel block. The inset shows the location of two selected electrodes. The t_1 to t_4 correspond to t_1 to t_4 in Fig. 5E, meaning the steel block is (t_1) sliding, (t_2) clamped, (t_3) placed on the ground, and (t_4) released.

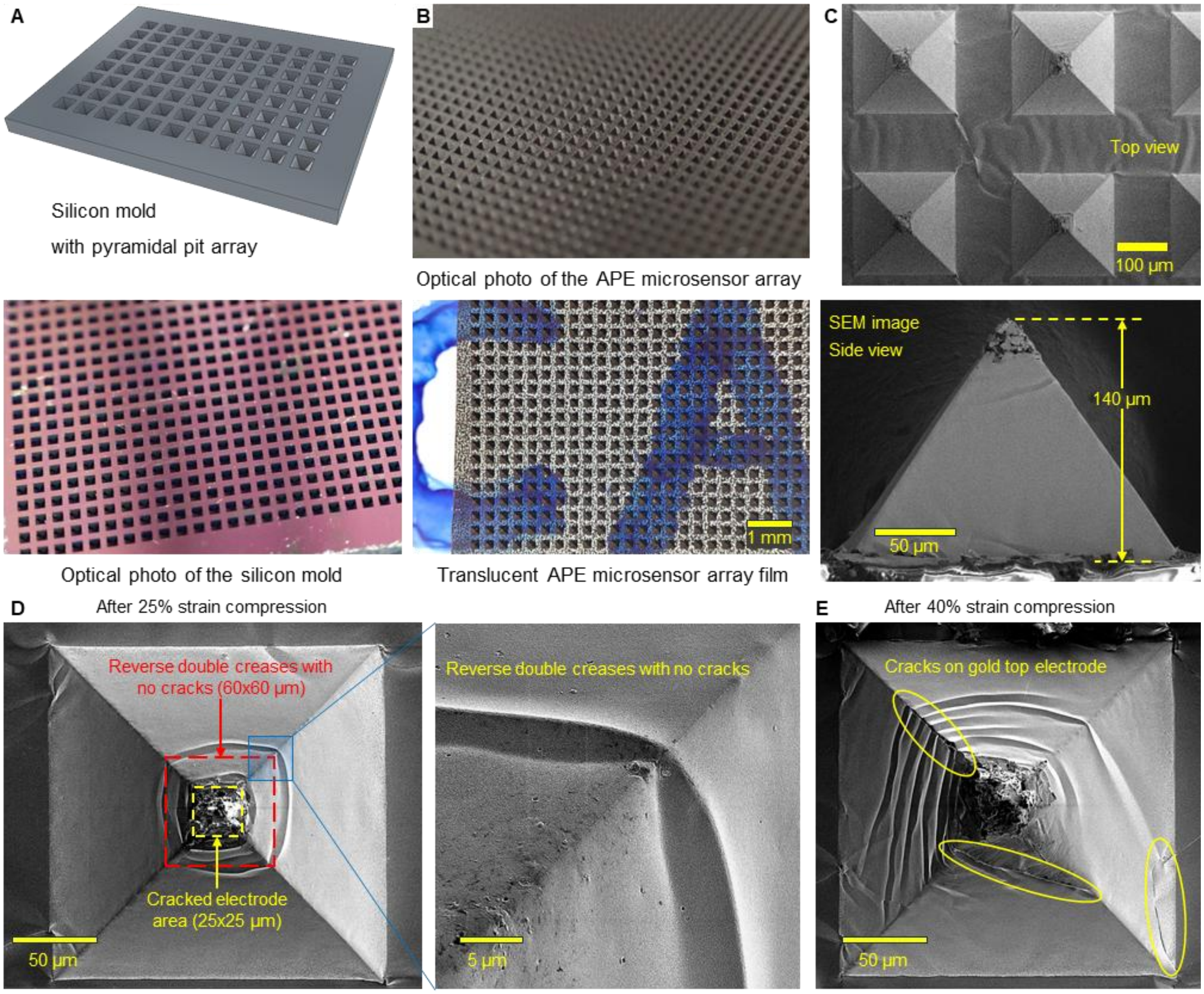

Supplementary Fig. 47.

Preparation of the APE microsensor array film. (**A**) 3D schematic diagram and optical photo of the silicon mould for curing microsensor array film. (**B**) Optical photo of the microsensor array film with a unit side length of 200 μm. (**C**) SEM images of the APE microsensor array deposited with 100 nm gold layer as the top electrode. (**D** and **E**) SEM images of an APE microsensor unit deposited with 100 nm gold layer as the top electrode. SEM images of APE microsensor units with deposited gold top electrodes after (**D**) 25% and (**E**) 40% compression deformation.

The APE microsensor array film has pyramid sensor units with a side length of 200 μm and a space of 100 μm, exhibiting a spatial resolution of 300 μm. This resolution is sufficient for tactile

applications in robots where the object sizes are typically above 1 mm. Each pyramid sensor is 140 µm high and the bottom layer of the microsensor film is 100 µm thick. Because the Ni particles are aligned along the magnetic field direction perpendicular to the film surface, the film is translucent along this direction (Supplementary Fig. 47B). This also facilitates the alignment of the microsensor film and the patterned electrodes on the glass wafer. After aligning the microsensor array and the bottom patterned electrodes, a 100 nm gold layer was deposited on top of the microsensor array film as the top electrode. Each sensor unit is subjected only to forces acting on the tip, rather than bending moments or torques. For example, the torque acting on the sensor array is decomposed into tangential forces on each sensor unit. It can be calculated from the curl of the tangential force field measured by the sensor array. This decomposition of complex loads is also one of the advantages of the array design.

Supplementary Fig. 47C show the top and side view of the microsensor unit. Within the designed pressure detection range, the maximum compressive strain of the sensor unit is approximately 25% (corresponding to a normal force of about 11 mN), which does not damage the porous composite material itself. However, it can cause damage to the gold coating at the contact points. Therefore, we applied a thicker gold electrode (30 nm) to the surface of the sensor units to make it more durable and coated it with an Ecoflex protective layer. Without the Ecoflex layer, the gold coating at the sensor tip cracks (approximately 25x25 µm area, 1.6% area of the top electrode) after a 25% strain compression. At 2/3 to 3/4 of the sensor height, around 30 µm from the tip, a ring of gold coating shows reverse double creases but no cracks, as seen in the SEM image in Supplementary Fig. 47D. This deformation pattern is consistent with that of thin-shell pyramid under compression. It is important to note that even if the gold electrode coating at the tip cracks, the sensor still functions normally. This is because the tip is only used for contact to conduct force and does not carry electrical current; the side and bottom electrodes remain intact and can accurately detect the pressure distribution on the bottom surface (see the current density simulation results inside the sensor unit in Supplementary Fig. 26). Compression tests under oblique loading also show the mechanical stability of the gold coating. At pressures five times higher than the design range (corresponding to approximately 40% compressive strain), the gold coating shows visible cracks, as shown in the SEM image in Supplementary Fig. 47E. At this point, the sensor exhibits significantly increased static resistance but still operates. To further protect the gold coating under mechanical loads, we coated the sensor array membrane with an Ecoflex layer after gold electrode deposition, which further prevents mechanical damage to the gold coating. This Ecoflex layer

allows loads applied at any position to be detected by adjacent sensor units, providing a spatially continuous force distribution with 200 μm resolution.

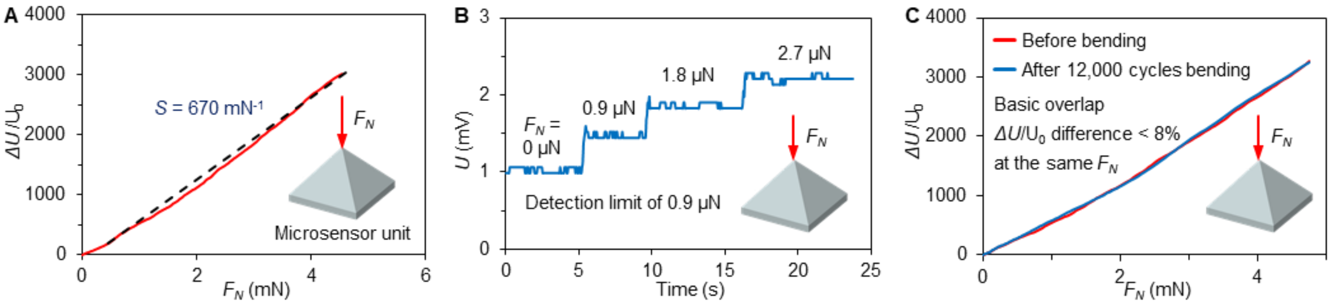

Supplementary Fig. 48.

Force sensing performance of a single APE microsensor unit with a side length of 200 μm. (**A**) Average relative potential change-force curve measured by four bottom electrodes of the APE microsensor unit. (**B**) The average potential measured by the four bottom electrodes of the microsensor unit under normal forces of 0, 0.9, 1.8, and 2.7 μN. (**C**) Average relative potential change-force curves measured by four bottom electrodes of the APE microsensor unit before and after 12,000 bending cycles.

We applied a normal force load on the microsensor unit and measured the output potentials of its four bottom electrodes. Supplementary Fig. 48A gives the average potential versus normal force curve, showing a high force sensitivity of 670 mN^{-1}. To measure the detection limit of the microsensor unit, we prepared 0.8×0.8×0.6 mm PDMS blocks as weights. The PDMS weight is stably placed on the four micro-sensor units, and each sensor unit is subject to a normal force of 0.9 μN when one weight is placed. We recorded the change in the average potential of the sensor bottom electrodes when weights were gradually added to the microsensor array, as shown in Supplementary Fig. 48B. It can be seen that the sensor can accurately detect a single PDMS block's weight, showing an ultra-low detection limit of 0.9 μN.

When mounted on a soft robot, the microsensor array film could deform along with the substrate. This deformation can be primarily borne by the composite film between sensor units, rather than the pyramid-shaped sensor units themselves. However, when the radius of curvature is below approximately 5 mm (corresponding to about 5% surface strain in the 0.5 mm-thick film), the vapor-deposited gold electrodes on the surface of the sensor array can crack, causing an open circuit. We performed long-term bending tests on the sensor array mounted on a 2 mm-thick flexible PDMS substrate. The maximum curvature in each bending cycle is 0.08 mm^{-1}, corresponding to 2% surface

strain of the sensor film. Results in Supplementary Fig. 48C show that before and after 12,000 bending cycles, the maximum relative output voltage variation of sensor units under the same load was < 8%, proving the long-term stability of the sensors.

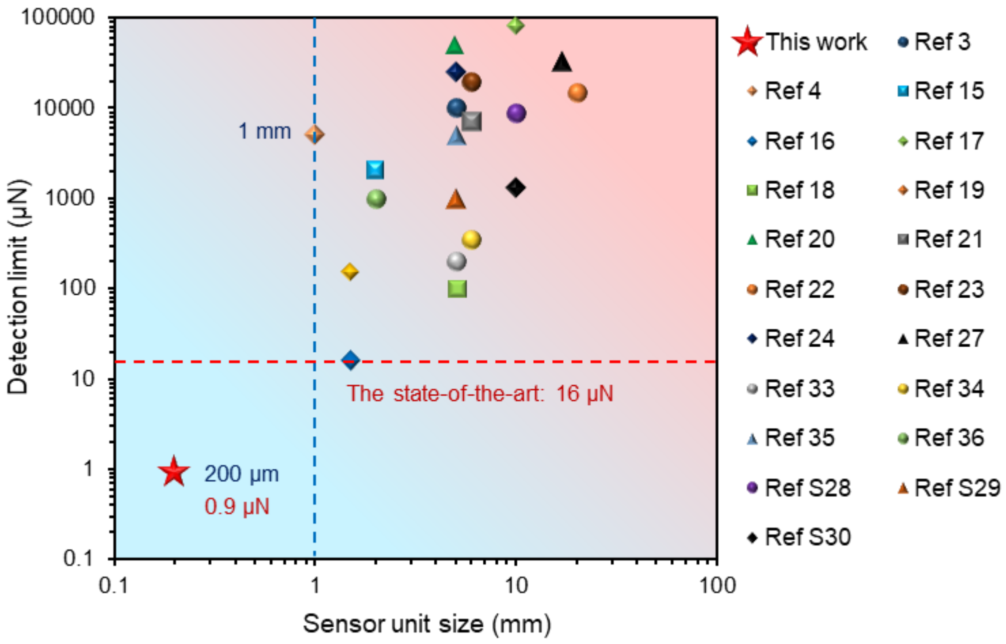

Supplementary Fig. 49.

Comparison of dimensions and detection limits between the APE sensor and state-of-the-art 3D force sensors(*2, 4, 15-24, 27, 33-36, S28-S30*).

Compared with existing 3D force sensors, APE exhibits not only the smallest size (200 μm), but also the lowest detection limit (0.9 μN). Most 3D force sensors show dimensions exceeding 5 mm, and even the smallest sensor (1 mm)(*4*) is one-order-of-magnitude larger than APE microsensors. In addition, the extremely low detection limit of APE microsensor (0.9 μN) is two-order-of-magnitude lower than state-of-the-arts (20 μN)(*16*).

Note that 200 μm is not the limit of miniaturisation for APE sensors. By increasing the mixing speed during the preparation process to reduce the size of FLG sheets and LM droplets to < 20 um, the sensor unit can achieve sizes below 50 μm. The main limitation to miniaturisation to that level at prototype stage is patterned electrode fabrication and alignment. Hence, we made it with 200 um sides.

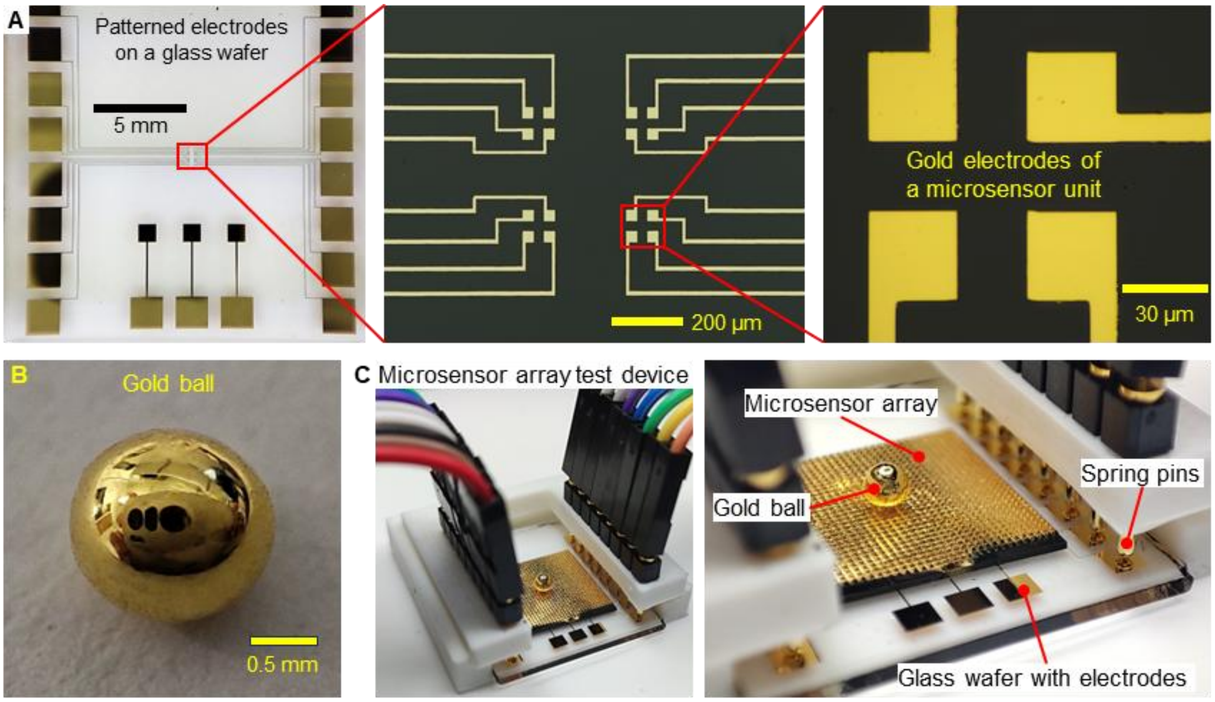

Supplementary Fig. 50.

Demonstration of APE microsensor array with sensor unit side length of 200 µm. (**A**) Photolithographically patterned electrodes on the glass wafer. (**B**) A gold ball prepared by melt solidification method. (**C**) experimental setup for metal ball detection test.

We use a laser writer and electron beam evaporator to create patterned gold electrodes on a glass wafer (Supplementary Fig. 50A). The electrodes distribution is as shown in Supplementary Fig. 25. Then gold and indium balls are placed on the microsensor film to demonstrate its 3D force detection capabilities. Gold balls are made by melting then solidifying gold particles on the evaporation boat in a thermal evaporator (Supplementary Fig. 50B). Supplementary Fig. 50C shows photos of the experimental setup. The microsensor array film is aligned and bonded with the patterned electrodes on the glass wafer under a microscope after plasma treatment. A 3D printed bracket is used to hold the spring pin connectors to connect the microsensor array with the Arduino UNO board. The Arduino UNO board calculates the force and direction on the four sensor units in contact with the metal ball in real time.

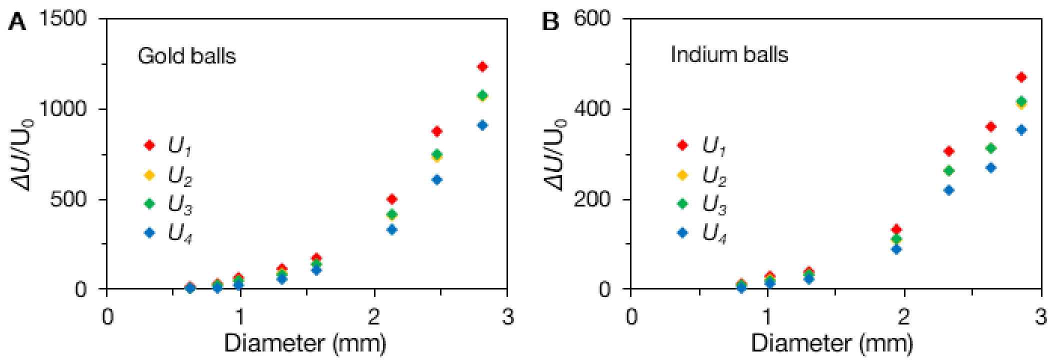

Supplementary Fig. 51.

Raw relative potential data measured by the bottom electrodes of the microsensor unit under (**A**) gold and (**B**) indium balls of different diameters. See the definition of force direction and U_1 ~ U_4 in Fig. 4A.

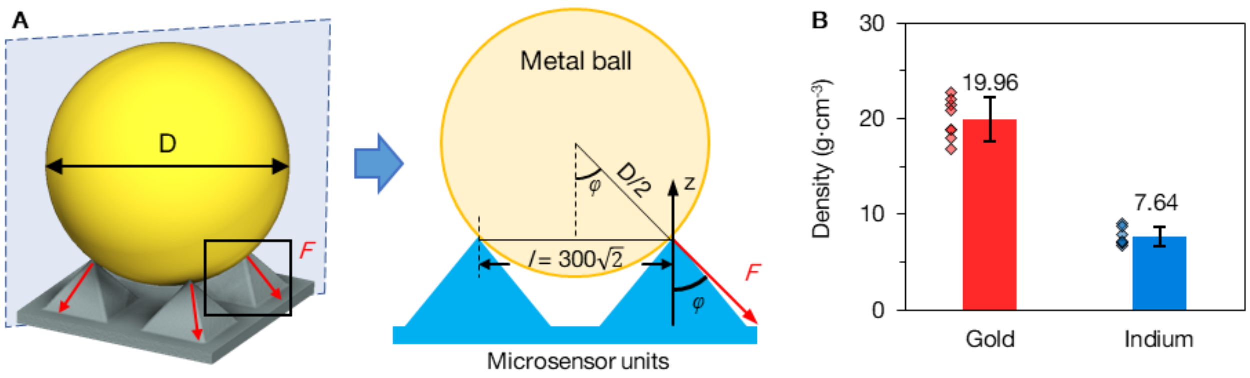

Supplementary Fig. 52.

(**A**) Force analysis of the microsensor unit under the metal ball. (**B**) Density calculated from on the mass and diameter of gold and indium metal balls measured by the microsensor array. For both error bars, n = 8 derived from different APE microsensor arrays, data are presented as mean values +/- SD.

When placed on the microsensor array film, the metal balls come into contact with the vertices of the four microsensor units, generating tilting forces. Due to the symmetrical distribution of microsensor units, the theoretical normal force on a single sensor unit is one-quarter of the weight of the metal ball. The direction of the shear force remains unchanged but its value depends on the diameter of the metal ball. Taking the sensor unit in the first quadrant marked in Supplementary Fig. 52A as an example, its shear force direction θ is 45° (see the measured shear force direction in Fig. 5K). Assuming that the force on the sensor unit is perpendicular to the surface of the metal ball, the angle φ between the force and the z-axis satisfies:

$$\sin(\varphi) = \frac{l/_2}{D/_2} = \frac{424.3}{D} \tag{S5}$$

Where D is the diameter of the metal ball, and l is the horizontal distance between the vertices of the two diagonally opposite sensor units. Since the distance between adjacent sensor vertices is 300 μm, l is around 424.3 μm.

Considering the friction force, the actual force direction φ will be smaller, but it still basically satisfies that the sine of φ is inversely proportional to the diameter of the metal ball, as proven in Fig. 5J. Therefore, the microsensor array can calculate the mass and diameter of the metal ball through the measured normal force and force direction respectively, and further identify the metal type through the calculated density, as shown in Supplementary Fig. 52B. The calculated densities of gold and indium

balls are 19.96 $g \cdot cm^{-3}$ and 7.64 $g \cdot cm^{-3}$, respectively, with a difference of <5% from their real density (19.3 $g \cdot cm^{-3}$ of gold and 7.31 $g \cdot cm^{-3}$ of indium).

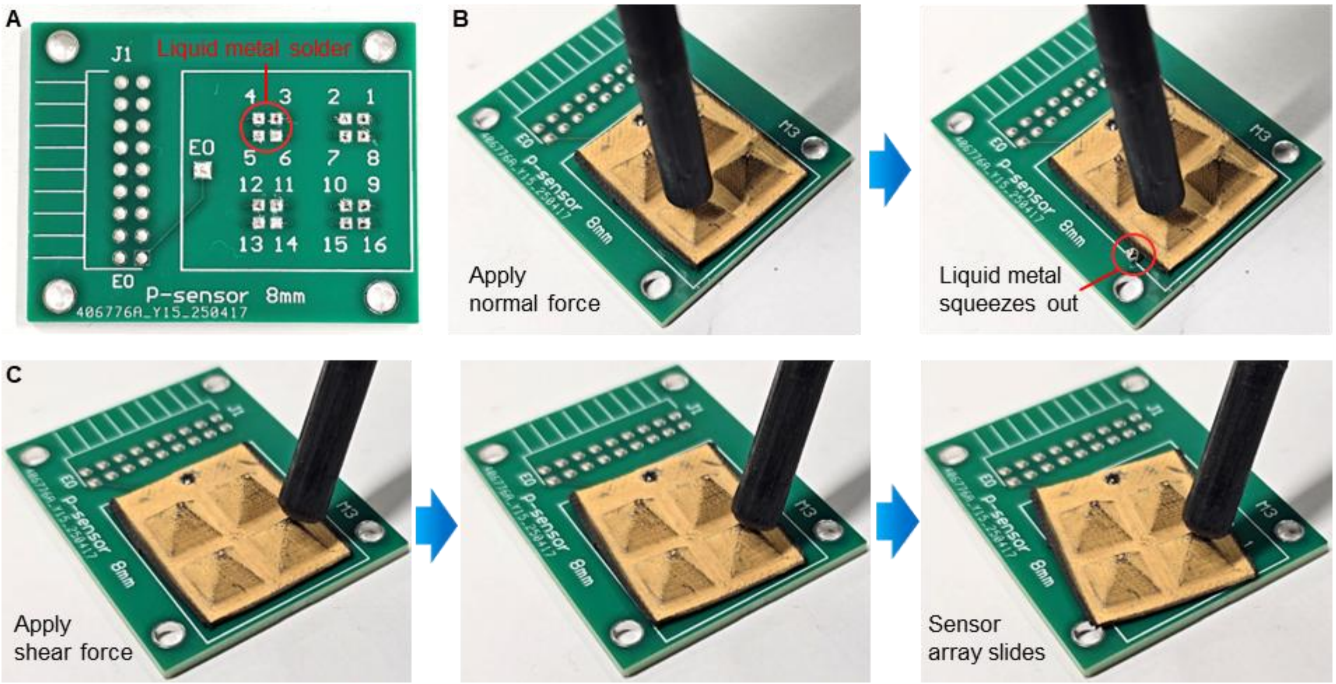

Supplementary Fig. 53.

Sensor array PCB connection based on liquid metal (EGaIn) solder. (**A**) PCB with liquid metal solder. (**B**) Liquid metal is extruded under pressure. (**C**) The sensor array slides under tangential force.

In the APE sensors, the chromium interlayer markedly improves interfacial adhesion between the gold electrodes and the composite substrate. Within the linear measurement range, the sensor units primarily experience vertical compression normal to the bottom surface, with minimal lateral deformation at the base. The chromium-gold double-layer electrode maintains stable electrical contact between APE sensors and the PCB during operation. Under excessive shear loading, detachment consistently occurs at the interface between the gold electrode and the Field's metal solder on PCB, not between the composite substrate and the chromium-gold electrode, confirming the interfacial robustness. Across all of the sensor array experiments, including 12,000 bending cycles shown in Supplementary Fig. 48, we observed no contact issues or interfacial failure.

Although LM electrodes such as EGaIn could improve compliance in principle, two critical limitations preclude their use in the APE sensor array. First, LM electrodes are highly prone to extrusion and leakage under pressure (Supplementary Fig. 53, A and B). This behaviour also prevents LM from maintaining stable boundaries, which increases the risk of short circuits between adjacent electrodes. This is particularly problematic in high-resolution APE sensor arrays. Second, in contrast to

Field’s metal solder that solidifies at room temperature, LM provides negligible adhesion to the PCB. This lack of bonding allows slippage of the sensor array under shear forces, compromising mechanical stability (Supplementary Fig. 53C).

Within the linear measurement range, the sensor unit primarily undergoes vertical compression perpendicular to the bottom surface when loaded, with no significant lateral deformation occurring at the base. The chromium interlayer also significantly enhances the interfacial strength between the gold electrode and the composite substrate. When excessive shear force causes the sensor unit to detach from the PCB, failure consistently occurs at the interface between the gold electrode and the Field’s metal solder, while the gold electrode itself remains well bonded to the sensor. During experiments, no contact issues or signal degradation resulting from interfacial failure between the composite and the gold electrode were observed. Furthermore, when deployed on flexible substrates, the gold electrodes in the sensor array can also withstand a certain degree of deformation. Even after 12,000 bending cycles in Supplementary Fig. 48, no response attenuation or contact issue was observed.

If liquid metal (LM) like EGaIn were used as a soft electrode, there would be two major issues. First, LM electrodes are highly prone to leakage under pressure due to extrusion (Supplementary Fig. 53, A and B). Additionally, liquid electrodes cannot maintain stable boundaries, easily leading to short circuits or poor contact between adjacent electrodes. Second, unlike Field’s metal solder, LM cannot provide adequate adhesion to secure the sensor array on the PCB, resulting in slippage under shear forces (Supplementary Fig. 53C).

References

S1. P. Anton, K. Uwe, M. Hartmut, P. Johann, In situ x-ray reflectivity study of the oxidation kinetics of liquid gallium and the liquid alloy. *J. Phys.: Condens. Matter* **10**, 971 (1998).

S2. Z. Tang, S. Jia, C. Zhou, B. Li, 3D printing of highly sensitive and large-measurement-range flexible pressure sensors with a positive piezoresistive effect. *ACS Appl. Mater. Interfaces* **12**, 28669–28680 (2020).

S3. M. Jian, K. Xia, Q. Wang, Z. Yin, H. Wang, C. Wang, H. Xie, M. Zhang, Y. Zhang, Flexible and Highly Sensitive Pressure Sensors Based on Bionic Hierarchical Structures. *Adv. Funct. Mater.* **27**, 1606066 (2017).

S4. R. Chen, T. Luo, J. Wang, R. Wang, C. Zhang, Y. Xie, L. Qin, H. Yao, W. Zhou, Nonlinearity synergy: An elegant strategy for realizing high-sensitivity and wide-linear-range pressure sensing. *Nat. Commun.* **14**, 6641 (2023).

S5. Y. Wei, S. Chen, Y. Lin, Z. Yang, L. Liu, Cu–Ag core–shell nanowires for electronic skin with a petal molded microstructure. *J. Mater. Chem. C* **3**, 9594-9602 (2015).

S6. P. Wei, X. Guo, X. Qiu, D. Yu, Flexible capacitive pressure sensor with sensitivity and linear measuring range enhanced based on porous composite of carbon conductive paste and polydimethylsiloxane. *Nanotechnology* **30**, 455501 (2019).

S7. S. Jung, J. H. Kim, J. Kim, S. Choi, J. Lee, I. Park, T. Hyeon, D.-H. Kim, Reverse-micelle-induced porous pressure-sensitive rubber for wearable human–machine interfaces. *Adv. Mater.* **26**, 4825-4830 (2014).

S8. Z. Sang, K. Ke, I. Manas-Zloczower, Design strategy for porous composites aimed at pressure sensor application. *Small* **15**, 1903487 (2019).

S9. Y.-F. Wang, T. Sekine, Y. Takeda, J. Hong, A. Yoshida, D. Kumaki, T. Shiba, S. Tokito, Deep eutectic solvent induced porous conductive composite for fully printed piezoresistive pressure sensor. *Adv. Mater. Technol.* **6**, 2100731 (2021).

S10. W. Huang, K. Dai, Y. Zhai, H. Liu, P. Zhan, J. Gao, G. Zheng, C. Liu, C. Shen, Flexible and lightweight pressure sensor based on carbon nanotube/thermoplastic polyurethane-aligned conductive foam with superior compressibility and stability. *ACS Appl. Mater. Interfaces* **9**, 42266-42277 (2017).

S11. B. Ji, Q. Zhou, B. Hu, J. Zhong, J. Zhou, B. Zhou, Bio-inspired hybrid dielectric for capacitive and triboelectric tactile sensors with high sensitivity and ultrawide linearity range. *Adv. Mater.* **33**, 2100859 (2021).

S12. J. Kim, M. Jang, G. Jeong, S. Yu, J. Park, Y. Lee, S. Cho, J. Yeom, Y. Lee, A. Choe, Y.-R. Kim, Y. Yoon, S. S. Lee, K.-S. An, H. Ko, MXene-enhanced β-phase crystallization in ferroelectric porous composites for highly-sensitive dynamic force sensors. *Nano Energy* **89**, 106409 (2021).

S13. J. Hwang, Y. Kim, H. Yang, J. H. Oh, Fabrication of hierarchically porous structured PDMS composites and their application as a flexible capacitive pressure sensor. *Composites, Part B* **211**, 108607 (2021).

S14. F.-R. Hsiao, I. F. Wu, Y.-C. Liao, Porous CNT/rubber composite for resistive pressure sensor. *J. Taiwan Inst. Chem. Eng.* **102**, 387-393 (2019).

S15. J. Yang, D. Tang, J. Ao, T. Ghosh, T. V. Neumann, D. Zhang, Y. Piskarev, T. Yu, V. K. Truong, K. Xie, Y.-C. Lai, Y. Li, M. D. Dickey, Ultrasoft liquid metal elastomer foams with positive and negative piezopermittivity for tactile sensing. *Adv. Funct. Mater.* **30**, 2002611 (2020).

S16. Y. Cheng, Y. Ma, L. Li, M. Zhu, Y. Yue, W. Liu, L. Wang, S. Jia, C. Li, T. Qi, J. Wang, Y. Gao, Bioinspired microspines for a high-performance spray $ti_3c_2t_x$ mxene-based piezoresistive sensor. *ACS Nano* **14**, 2145-2155 (2020).

S17. Y. Gao, C. Yan, H. Huang, T. Yang, G. Tian, D. Xiong, N. Chen, X. Chu, S. Zhong, W. Deng, Y. Fang, W. Yang, Microchannel-confined mxene based flexible piezoresistive multifunctional micro-force sensor. *Adv. Funct. Mater.* **30**, 1909603 (2020).

S18. J. C. Yang, J.-O. Kim, J. Oh, S. Y. Kwon, J. Y. Sim, D. W. Kim, H. B. Choi, S. Park, Microstructured porous pyramid-based ultrahigh sensitive pressure sensor insensitive to strain and temperature. *ACS Appl. Mater. Interfaces* **11**, 19472-19480 (2019).

S19. J.-K. Lee, H.-H. Kim, J.-W. Choi, K.-C. Lee, S. Lee, Development of direct-printed tactile sensors for gripper control through contact and slip detection. *Int. J. Control Autom. Syst.* **16**, 929-936 (2018).

S20. W. Liu, N. Liu, Y. Yue, J. Rao, F. Cheng, J. Su, Z. Liu, Y. Gao, Piezoresistive pressure sensor based on synergistical innerconnect polyvinyl alcohol nanowires/wrinkled graphene film. *Small* **14**, 1704149 (2018).

S21. Y. Xiong, Y. Shen, L. Tian, Y. Hu, P. Zhu, R. Sun, C.-P. Wong, A flexible, ultra-highly sensitive and stable capacitive pressure sensor with convex microarrays for motion and health monitoring. *Nano Energy* **70**, 104436 (2020).

S22. Z. Qiu, Y. Wan, W. Zhou, J. Yang, J. Yang, J. Huang, J. Zhang, Q. Liu, S. Huang, N. Bai, Z. Wu, W. Hong, H. Wang, C. F. Guo, Ionic skin with biomimetic dielectric layer templated from calathea zebrine leaf. *Adv. Funct. Mater.* **28**, 1802343 (2018).

S23. H. Xu, L. Gao, Y. Wang, K. Cao, X. Hu, L. Wang, M. Mu, M. Liu, H. Zhang, W. Wang, Y. Lu, Flexible waterproof piezoresistive pressure sensors with wide linear working range based on conductive fabrics. *Nano-Micro Lett.* **12**, 159 (2020).

S24. K. Bae, J. Jeong, J. Choi, S. Pyo, J. Kim, Large-area, crosstalk-free, flexible tactile sensor matrix pixelated by mesh layers. *ACS Appl. Mater. Interfaces* **13**, 12259-12267 (2021).

S25. J. He, P. Xiao, W. Lu, J. Shi, L. Zhang, Y. Liang, C. Pan, S.-W. Kuo, T. Chen, A Universal high accuracy wearable pulse monitoring system via high sensitivity and large linearity graphene pressure sensor. *Nano Energy* **59**, 422-433 (2019).

S26. X.-H. Zhao, Q.-T. Lai, W.-T. Guo, Z.-H. Liang, Z. Tang, X.-G. Tang, V. A. L. Roy, Q.-J. Sun, Skin-inspired highly sensitive tactile sensors with ultrahigh resolution over a broad sensing range. *ACS Appl. Mater. Interfaces* **15**, 30486-30494 (2023).

S27. Q.-J. Sun, X.-H. Zhao, Y. Zhou, C.-C. Yeung, W. Wu, S. Venkatesh, Z.-X. Xu, J. J. Wylie, W.-J. Li, V. A. L. Roy, Fingertip-skin-inspired highly sensitive and multifunctional sensor with hierarchically structured conductive graphite/polydimethylsiloxane foams. *Adv. Funct. Mater.* **29**, 1808829 (2019).

S28. Y.-F. Wang, J. Yoshida, Y. Takeda, A. Yoshida, T. Kaneko, T. Sekine, D. Kumaki, S. Tokito, Printed Composite Film with Microporous/Micropyramid Hybrid Conductive Architecture for Multifunctional Flexible Force Sensors. *Nanomaterials* **14**, 63 (2024).

S29. X. Zhou, L. Zhang, Y. Wang, S. Zhao, Y. Zhou, Y. Guo, Y. Wang, J. Liang, H. Chen, Aerosol jet printing of multi-dimensional oect force sensor with high sensitivity and large measuring range. *Adv. Mater. Technol.* **8**, 2201272 (2023).

S30. H. Dai, C. Zhang, C. Pan, H. Hu, K. Ji, H. Sun, C. Lyu, D. Tang, T. Li, J. Fu, P. Zhao, Split-type magnetic soft tactile sensor with 3d force decoupling. *Adv. Mater.* **36**, 2310145. (2024).

Supplementary Video 1.

Real-time 3D force sensing of APE sensor unit.

Supplementary Video 2.

The A4 paper tube gripping demonstration of a robotic arm equipped with APE sensor array.

Supplementary Video 3.

The A4 paper tube gripping demonstration of a robotic arm equipped with a commercial force sensor.

Supplementary Video 4.

The steel block transferring demonstration of a robotic arm equipped with APE sensor array.

Supplementary Video 5.

The failure to detect the sliding of the steel block of a robotic arm equipped with a commercial force sensor.

Supplementary Video 6.

The steel block transferring demonstration of a robotic arm equipped with a commercial force sensor.
